# Central regulation of feeding and body weight by ciliary GPR75

**DOI:** 10.1172/JCI182121

**Published:** 2024-08-13

**Authors:** Yiao Jiang, Yu Xun, Zhao Zhang

**Affiliations:** 1 Center for the Genetics of Host Defense and; 2 Division of Endocrinology, Department of Internal Medicine, University of Texas Southwestern Medical Center, Dallas, Texas, USA.

**Keywords:** Cell biology, Metabolism, G protein&ndash;coupled receptors, Obesity

## Abstract

Variants of the G protein–coupled receptor 75 (*GPR75*) are associated with a lower BMI in large-scale human exome-sequencing studies. However, how GPR75 regulates body weight remains poorly understood. Using random germline mutagenesis in mice, we identified a missense allele (*Thinner*) of *Gpr75* that resulted in a lean phenotype and verified the decreased body weight and fat weight in *Gpr75*-knockout (*Gpr75^–/–^*) mice. *Gpr75^–/–^* mice displayed reduced food intake under high-fat diet (HFD) feeding, and pair-feeding normalized their body weight. The endogenous GPR75 protein was exclusively expressed in the brains of 3xFlag-tagged *Gpr75*-knockin (*3xFlag-Gpr75*) mice, with consistent expression across different brain regions. GPR75 interacted with Gα_q_ to activate various signaling pathways after HFD feeding. Additionally, GPR75 was localized in the primary cilia of hypothalamic cells, whereas the *Thinner* mutation (L144P) and human GPR75 variants in individuals with a lower BMI failed to localize in the cilia. Loss of GPR75 selectively inhibited weight gain in HFD-fed mice but failed to suppress the development of obesity in leptin *ob*–mutant (*Lep^ob^*-mutant) mice and adenylate cyclase 3–mutant (*Adcy3*-mutant) mice on a chow diet. Our data reveal that GPR75 is a ciliary protein expressed in the brain and plays an important role in regulating food intake.

## Abstract 2

GPR75 variants linked to lower BMI in humans; mouse study reveals its role in reducing weight by decreasing food intake, highlighting its significance as a brain-expressed ciliary protein regulating appetite.

## Introduction

The prevalence of obesity has become a major threat to human health (1). The development of obesity is a complex process determined by both genetics and the environment (2). It has become clear that an obesogenic environment increases the risk of obesity, while genetic factors play an important role in determining the susceptibility to obesity (3, 4). Mice are the most widely used animal model to study human obesity due to their physiologic similarity to humans. Since the positional cloning of the mouse obese (*ob*) gene (5), many studies with different mouse models have provided fundamental insights into the development of obesity. However, the genetic basis for obesity remains poorly understood, largely because of the complex physiological processes that lead to obesity. To identify new genes and pathways associated with obesity, we have adopted a *N*-ethyl-*N*-nitrosourea–based (ENU-based) phenotype-driven forward genetic screening coupled with automated meiotic mapping (AMM) in mice (6). Both body weight and body composition were measured in these mice carrying different mutations, which allowed us to identify both obese and lean phenotypes.

G protein–coupled receptor 75 (GPR75) was first identified in 1999 as a human orphan GPCR that maps to chromosome 2p16 (7). Studies have revealed that GPR75 is involved in insulin secretion and insulin signaling (8, 9), vascular function and hypertension (10, 11), neuroprotection (12), and prostate tumor metastasis (13). Recently, large-scale human exome sequencing identified a significant association of *GPR75* variants with lower BMI (14). Although as an orphan GPCR, it has been reported by different groups that both C-C motif chemokine ligand 5 (CCL5, also known as RANTES) (15) and 20-hydroxyeicosatetraenoic acid (20-HETE) (10, 16) are ligands for GPR75. However, the molecular mechanism of GPR75 in the regulation of body weight is poorly understood. Here, we describe a lean phenotype that was caused by a point mutation in *Gpr75* from our mouse screening. Furthermore, we explored the underlying cause of the phenotype and examined potential GPR75 ligands involved in regulating body weight.

## Results

### Identification of the Thinner allele.

**Figure 1:**
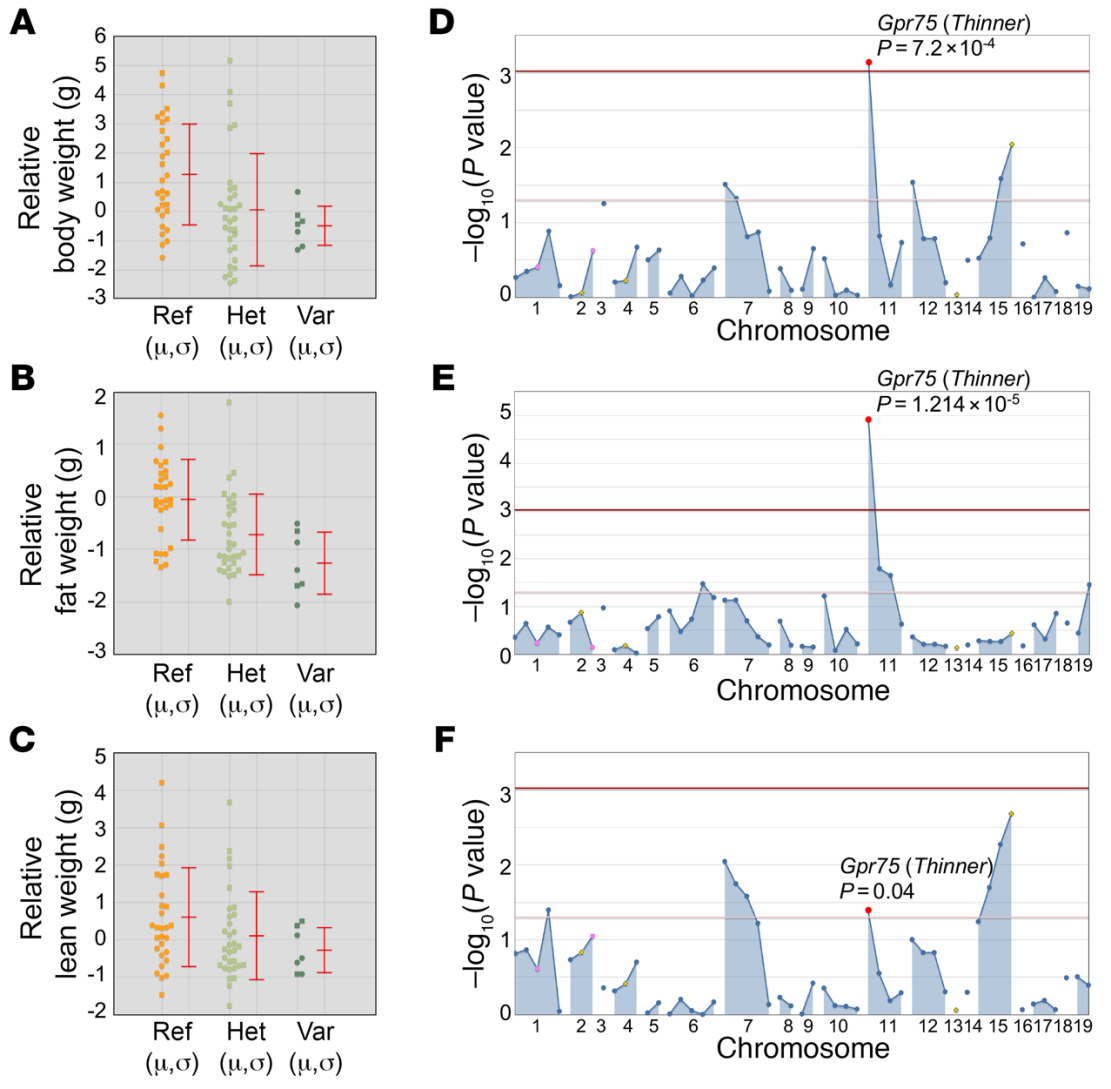
Identification and mapping of the *Thinner* allele. (**A**–**C**) Relative body weight (**A**), fat weight (**B**), and lean weight (**C**) phenotypic data plotted versus genotype at the *Gpr75* mutation site. Mean (μ) and SD (σ) are indicated. Ref, homozygous for the reference allele; Het, heterozygous for the reference allele and for the *Thinner* allele; Var, homozygous for the *Thinner* allele. Raw weight data were compared with the predicted weight of mice based on age and sex to calculate the relative values, minimizing the effects of age and sex differences in G3 mice. (**D**–**F**) Manhattan plots showing *P* values calculated using an additive model of inheritance about relative body weight (**D**), fat weight (**E**), and lean weight (**F**). The −log_10_ *P* values (*y* axis) are plotted versus the chromosomal positions of 53 mutations (*x* axis) identified in the G1 founder of the pedigree. Horizontal dark red and pink lines represent thresholds of *P* = 0.05 with and without Bonferroni’s correction, respectively.

The *Thinner* phenotype is observed among third-generation (G3) C57BL/6J mice heterozygous or homozygous for mutations induced by ENU. *Thinner* mice have decreased body weight, decreased fat mass, and slightly decreased lean mass compared with WT mice (Figure 1, A–C). The *Thinner* phenotype was mapped as a quantitative trait. AMM (6) implicated a missense allele of *Gpr75* as the causative mutation, displaying the strongest linkage in an additive model of inheritance (Figure 1, D–F). The *Thinner* mutation was a single nucleotide transition from T to C, causing substitution of a lysine for a proline at position 144 (L144P) in the GPR75 protein (Supplemental Figure 1A; supplemental material available online with this article; https://doi.org/10.1172/JCI182121DS1). These data suggest an association between GPR75 and leanness in mice.

### GPR75-deficient mice exhibit a lean phenotype.

**Figure 2:**
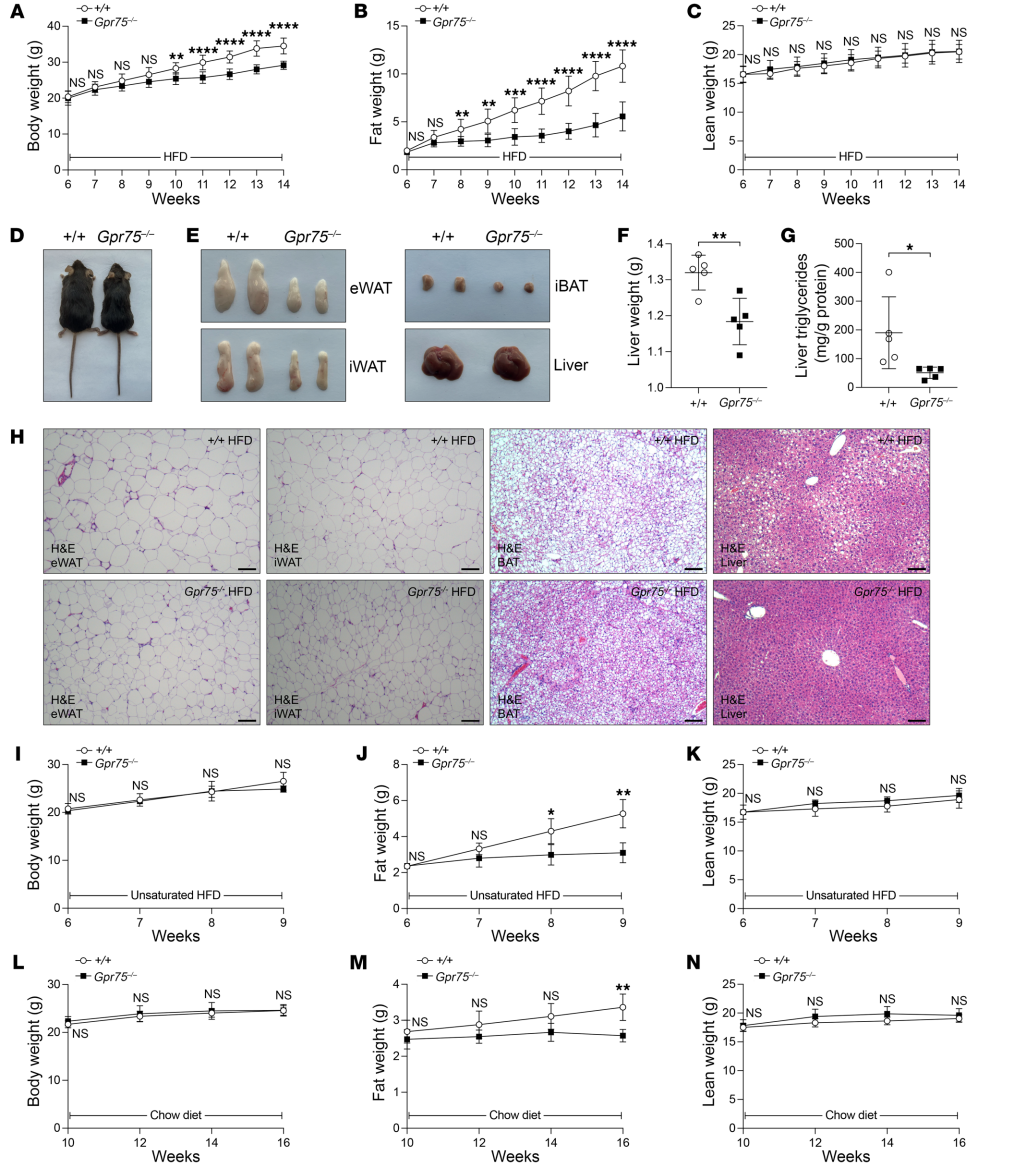
The phenotype of *Gpr75^–/–^* mice. (**A**–**C**) Body weight (**A**), fat weight (**B**), and lean weight (**C**) of male *Gpr75^–/–^* mice (*n* = 9) and WT littermates (*n* = 9) fed a HFD from 6 to 14 weeks of age. (**D**) Photograph of a 14-week-old male *Gpr75^–/–^* mouse and a WT (+/+) littermate fed a HFD for 8 weeks. (**E**) Representative photographs of eWAT, iWAT, iBAT, and liver from 14-week-old male mice fed a HFD for 8 weeks. (**F** and **G**) Liver weight (**F**) and liver triglyceride levels (**G**) of 14-week-old male mice fed a HFD for 8 weeks. (**H**) H&E stainings of sections from eWAT, iWAT, iBAT, and liver of 14-week-old male mice fed a HFD for 8 weeks. Scale bars: 100 μm. (**I**–**K**) Body weight (**I**), fat weight (**J**), and lean weight (**K**) of male *Gpr75^–/–^* mice (*n* = 5) and WT littermates (*n* = 5) fed an unsaturated HFD from 6 to 9 weeks of age. (**L**–**N**) Body weight (**L**), fat weight (**M**), and lean weight (**N**) of male *Gpr75^–/–^* mice (*n* = 7) and WT littermates (*n* = 7) fed a chow diet from 10 to 16 weeks of age. Data are presented as the mean ± SD. *P* values were determined by a mixed-effects model with Holm-Šidák’s multiple-comparison test (**A**–**C** and **I**–**N**) or 2-tailed, unpaired Student’s *t* test (**F** and **G**). *P ≤ 0.05, **P ≤ 0.01, ***P ≤ 0.001, and ****P ≤ 0.0001; NS, *P* > 0.05. Data points represent individual mice (**F** and **G**). Data are representative of 2 independent experiments.

The *Thinner* mutation (L144P) did not affect the stability of GPR75 as revealed by expression levels similar to those in the WT protein in 293T cells (Supplemental Figure 1B). We suspected that the mutation affects the function of GPR75 protein. By CRISPR/Cas9 gene targeting, we introduced a null allele of *Gpr75*, encoding the first 6 aa followed by 9 aberrant aa and a termination codon, into the germline of *C57BL/6J* mice (Supplemental Figure 1, A and C). Homozygous *Gpr75*-knockout (*Gpr75^–/–^*) mice had no reproductive or developmental defects, and both male and female *Gpr75^–/–^* mice were fertile. There was no difference in body weights, fat weights, or lean weights between WT and *Gpr75^–/–^* mice at 6 weeks of age (Figure 2, A–C). However, after only 2 weeks of HFD feeding, *Gpr75^–/–^* mice began to exhibit significantly decreased fat weight compared with WT mice (Figure 2B). After 4 weeks of HFD feeding, *Gpr75^–/–^* mice had decreased body weight (Figure 2A). No difference in lean weight was observed between WT and *Gpr75^–/–^* mice over the course of 8 weeks of HFD feeding (Figure 2C). The lean phenotype of *Gpr75^–/–^* mice fed a HFD for 8 weeks was obvious, with a smaller size of epididymal white adipose tissue (eWAT), interscapular white adipose tissue (iWAT), interscapular brown adipose tissue (iBAT), and liver (Figure 2, D and E). Additionally, *Gpr75^–/–^* mice had decreased liver weight and liver triglycerides (Figure 2, F and G). H&E staining further revealed smaller adipocyte size in the adipose tissues and reduced fat content in the liver of *Gpr75^–/–^* mice (Figure 2H). The HFD used in our study contained high levels of saturated fatty acids (lard), which are known to rapidly induce obesity in susceptible C57BL/6J mice. To explore whether different types of fatty acids influence the phenotype of *Gpr75^–/–^* mice, we fed mice a HFD rich in unsaturated fatty acids (safflower oil). Interestingly, *Gpr75^–/–^* mice also exhibited decreased fat weight compared with WT mice, with no change in body weight or lean weight (Figure 2, I–K). Unlike on a HFD, *Gpr75^–/–^* mice on a regular chow diet displayed very small body weight change and fat weight change compared with WT mice. Only a slight difference was observed in the fat weight of WT and *Gpr75^–/–^* mice at 16 weeks of age (Figure 2, L–N).

To further check other metabolic profiles of *Gpr75^–/–^* mice, we measured the fasting serum of WT and *Gpr75^–/–^* mice after 4 weeks of HFD feeding. We observed no difference in blood glucose, insulin, cholesterol, or triglyceride levels between WT and *Gpr75^–/–^* mice (Supplemental Figure 2, A–D). However, *Gpr75^–/–^* mice had significantly decreased levels of leptin, which was likely caused by the decreased fat mass (Supplemental Figure 2E). Both the glucose tolerance test (GTT) and insulin tolerance test (ITT) were performed on WT and *Gpr75^–/–^* mice, and no notable differences were observed (Supplemental Figure 2, F and G). These data suggest that there was no change in glucose or insulin metabolism between WT and *Gpr75^–/–^* mice at the onset of the lean phenotype development.

### The lean phenotype of Gpr75^–/–^ mice is due to decreased food intake.

**Figure 3:**
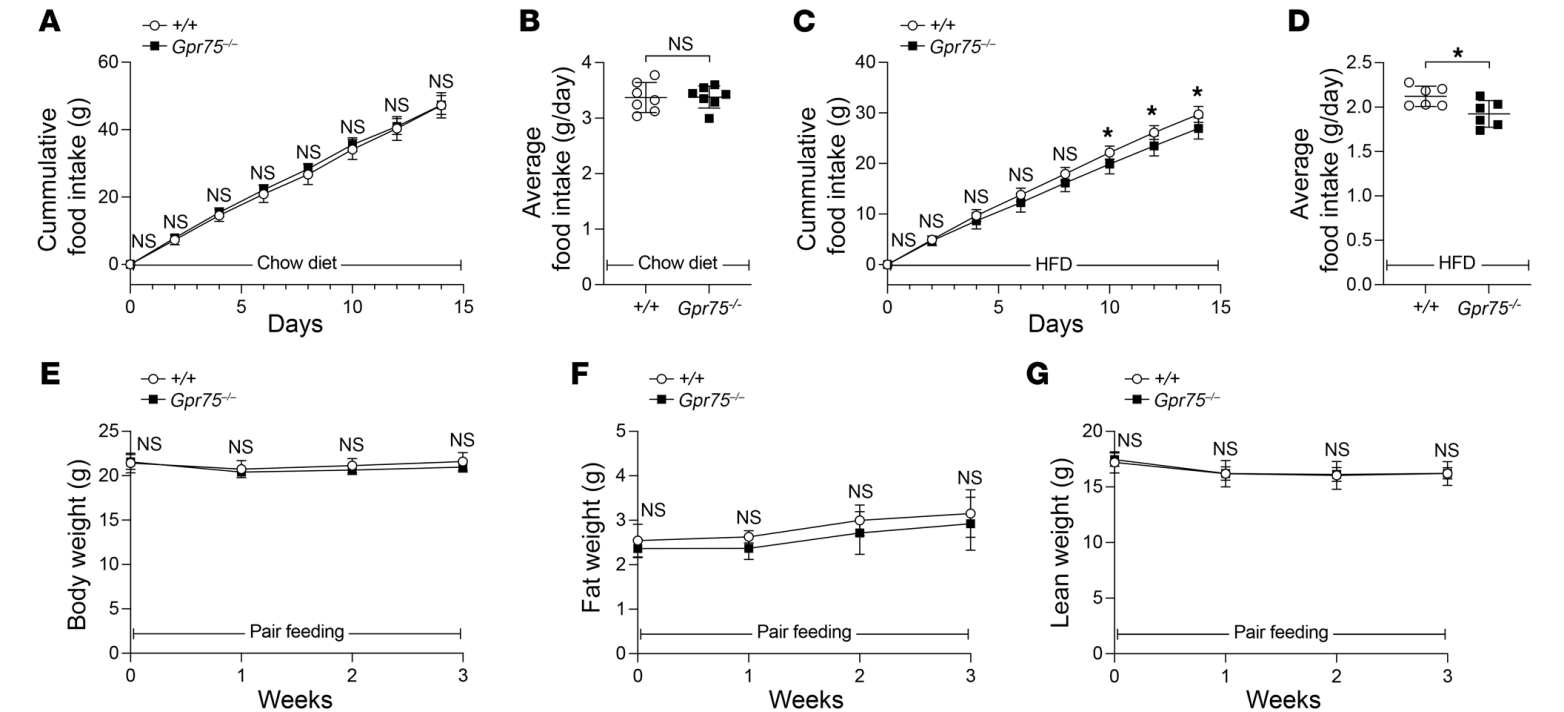
*Gpr75^–/–^* mice have a decrease in food intake, which causes the lean phenotype. (**A** and **B**) Food intake of male *Gpr75^–/–^* mice (*n* = 7) and WT littermates (*n* = 7) on a chow diet was monitored from 6 to 8 weeks of age. Cumulative food intake (g) (**A**) and average food intake per mouse per day (g/day) during the 2-week period (**B**). (**C** and **D**) Food intake of male *Gpr75^–/–^* mice (*n* = 6) and WT littermates (*n* = 6) fed a HFD was monitored from 6 to 8 weeks of age. Cumulative food intake (g) (**C**) and average food intake per mouse per day (g/day) during the 2-week period (**D**). (**E**–**G**) Body weight (**E**), fat weight (**F**), and lean weight (**G**) of male *Gpr75^–/–^* mice (*n* = 5) and WT littermates (*n* = 5) before and after pair-feeding from 6 weeks of age. Data are presented as the mean ± SD. *P* values were determined by 2-tailed, unpaired Student’s *t* test (**A**–**D**) or a mixed-effects model with Holm-Šidák’s multiple-comparison test (**E**–**G**). *P ≤ 0.05; NS, *P* > 0.05. Data points represent individual mice (**B**, **D**), and data are representative of 2 independent experiments.

Food intake of WT and *Gpr75^–/–^* mice on a chow diet or a HFD was monitored beginning at 6 weeks of age (Figure 3, A–D). On the chow diet, we noted no difference in food intake between *Gpr75^–/–^* mice and WT littermates (Figure 3, A and B). However, food intake was significantly decreased in *Gpr75^–/–^* mice compared with WT mice after HFD feeding for 10 days (Figure 3, C and D). To check if the decreased food intake was the cause of the lean phenotype in GPR75-deficient mice, WT mice were pair-fed with the same amount of a HFD as *Gpr75^–/–^* mice beginning at 6 weeks of age when the body weights, fat weights, and lean weights of the mice were similar (Figure 3, E–G). Three weeks after pair-feeding with a HFD, WT and *Gpr75^–/–^* mice gained similar amounts of body weight, fat weight, and lean weight (Figure 3, E–G), implying that the decreased food intake directly contributed to the development of the lean phenotype in GPR75-deficient mice.

To check whether there were other factors beyond the decreased food intake that contributed to the lean phenotype of GPR75-deficient mice, we performed metabolic cage experiments on WT and *Gpr75^–/–^* mice after 2 weeks of HFD feeding (Supplemental Figure 3). At this stage, *Gpr75^–/–^* mice exhibited decreased body weights and fat weights but similar lean weights compared with WT mice (Supplemental Figure 3, A–C). No significant differences were observed in energy expenditure, respiratory exchange ratio, or physical activities between WT and *Gpr75^–/–^* mice (Supplemental Figure 3, D–I). Furthermore, *Gpr75^–/–^* mice also demonstrated a comparable ability to maintain body temperature compared with WT mice during an acute cold stress experiment without food (Supplemental Figure 3J). To eliminate the effect of difference in food intake on the metabolic cage experiments, we pair-fed WT mice the same amount of a HFD as *Gpr75^–/–^* mice every day when conducting the metabolic cage experiments. Despite this standardization, we observed no differences between WT and *Gpr75^–/–^* mice (Supplemental Figure 4, A–I). Additionally, *Gpr75^–/–^* mice showed no deficiencies in food digestion and absorption as revealed by a similar fecal energy density compared with that of WT mice (Supplemental Figure 4J). Taken together, these findings strongly suggest that reduced food intake is the sole determinant contributing to the lean phenotype observed in GPR75-deficient mice.

### GPR75 is predominantly expressed in the brain and interacts with Gα_q_ to signal in the brain.

**Figure 4:**
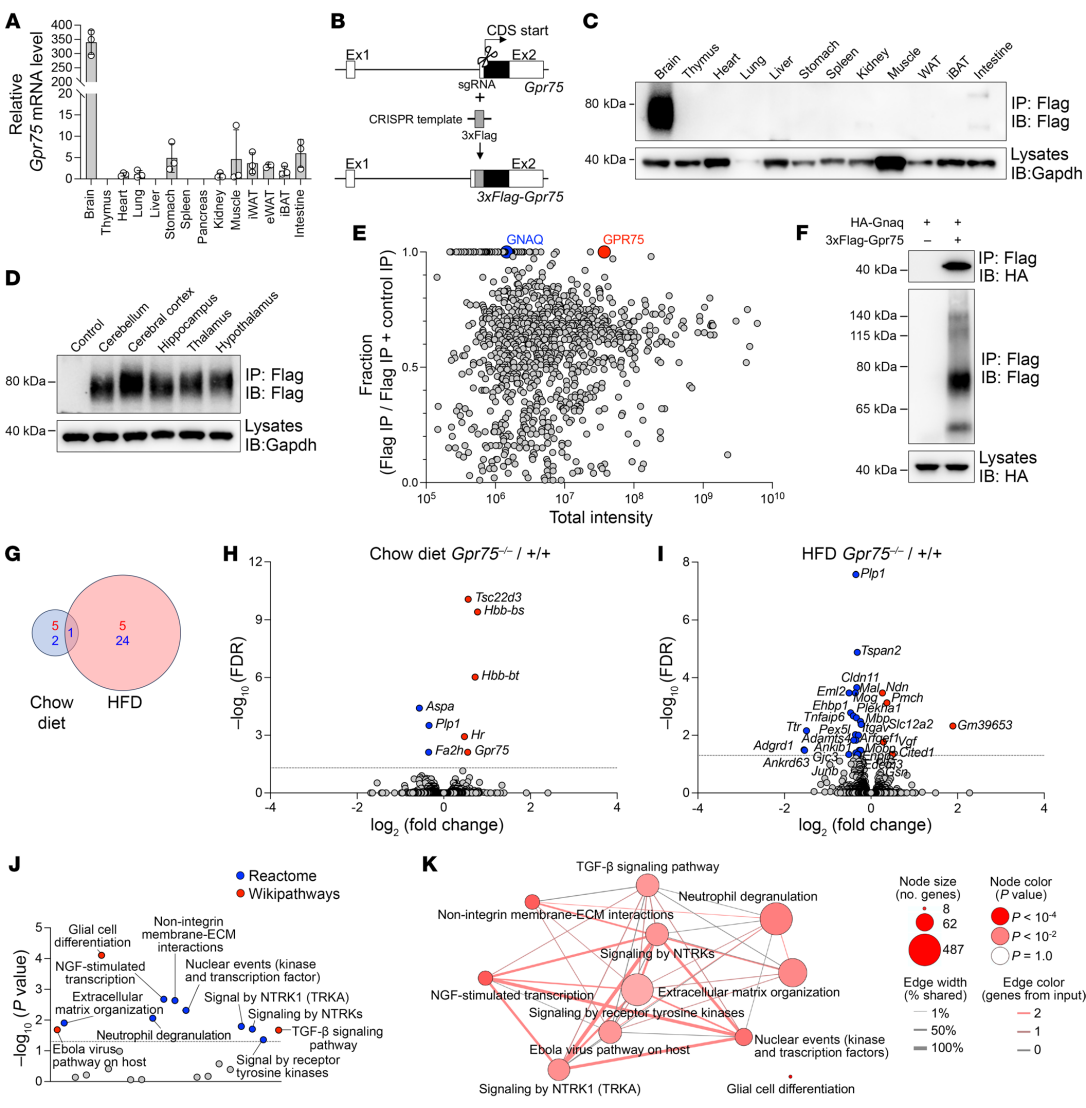
GPR75 is highly expressed in the brain and directly binds to Gα_q_ to regulate various downstream signaling pathways. (**A**) Relative *Gpr75* mRNA levels in different mouse tissues normalized by *Polr2a* (*n* = 3 mice). (**B**) Generation of 3xFlag-tagged Gpr75-knockin mice by CRISPR. Ex1, exon 1; Ex2, exon 2. (**C**) Immunoblot analysis of 3xFlag-Gpr75 protein expression in different mouse tissues (8-week-old males) by immunoprecipitation (IP). GAPDH was used as a loading control. IB, immunoblot. (**D**) Immunoblot analysis of 3xFlag-Gpr75 protein expression in various brain regions (8-week-old males) by immunoprecipitation. GAPDH was used as a loading control. (**E**) Mass spectrometric identification of GPR75-interacting proteins from Flag immunoprecipitates of Gpr75-3xFlag–knockin brain lysates. (**F**) Immunoblot analysis of immunoprecipitates (top and middle) and lysates (bottom) of 293T cells expressing HA-tagged Gnaq and 3xFlag-tagged Gpr75. (**G**) Summary of significantly changed genes (FDR <0.05) from RNA-Seq of hypothalamus from 8-week-old male WT and *Gpr75^–/–^* mice fed a chow diet or a HFD for 2 weeks (*n* = 3 mice for each group). (**H** and **I**) Volcano plots of differentially expressed genes in the hypothalamus of *Gpr75^–/–^* versus WT mice on a chow diet (**H**) or a 2-week HFD (**I**). Differentially expressed genes (FDR <0.05) are colored in red and blue indicating upregulation and downregulation, respectively. (**J**) Manhattan-like plot of pathways significantly associated (*P* < 0.05) with the loss of GPR75 on HFD feeding identified from a pathway overrepresentation analysis that mapped significant genes to the Reactome and WikiPathways databases. (**K**) Visualization of significantly associated pathways (*P* < 0.05) from **J**. Data are presented as the mean ± SD. Data points represent individual mice in **A**, and data are representative of 3 independent experiments.

To understand why GPR75-deficient mice experienced reduced food intake, we examined the mRNA levels of *Gpr75* in various mouse tissues. The expression of *Gpr75* mRNA was notably higher in the brain compared with all other tested tissues, indicating a potential role of GPR75 in the brain (Figure 4A). Using the single-cell RNA-Seq (scRNA-Seq) data (17) from the Allen Brain Cell Atlas, we thoroughly analyzed the expression of *Gpr75* mRNA in different cells of the mouse brain. Among 4.04 million brain cells, *Gpr75* was expressed in 0.327 million cells, accounting for 8.09% of all cells in the brain (Supplemental Figure 5A). The majority of *Gpr75*^+^ cells belong to neuronal classes (88.68%), with only small portions represented by granule and immature neuronal classes (4.57%) and non-neuronal classes (6.75%) (Supplemental Figure 5, B and C). *Gpr75*^+^ cells were widely distributed among all neuronal classes and various brain regions, with a relatively higher percentage among serotonergic neurons (36.60%) (Supplemental Figure 5, B–G).

To check the relative expression of endogenous GPR75 protein, we generated *3xFlag-Gpr75*–knockin mice using CRISPR-mediated homologous replacement (Figure 4B). The endogenous GPR75 protein expression was exclusively detected in the Flag immunoprecipitates of brain lysates, aligning with the *Gpr75* mRNA expression profile (Figure 4C). Different parts of the brain have diverse functions, and the hypothalamus plays a crucial role in the regulation of food intake. Hence, we evaluated the expression of endogenous GPR75 protein in various brain regions. Consistent with the scRNA-seq data (Supplemental Figure 5, D and E), we found that GPR75 protein was expressed throughout different parts of the brain without clear regional differences (Figure 4D).

To investigate the molecular mechanism underlying the role of GPR75 in the regulation of food intake, we utilized our *3xFlag-Gpr75*–knockin mice to pull down GPR75 in brain lysates for the identification of interacting proteins by mass spectrometry (Figure 4E). GNAQ (aka Gαq), a guanine nucleotide–binding protein, was identified as a GPR75-interacting protein (Figure 4E). We further confirmed the interaction between GPR75 and Gα_q_ by immunoprecipitation assays (Figure 4F). Prior studies have indicated that Gα_q_ mediates GPR75 signaling (15). Thus, it is plausible that GPR75 also functions through Gα_q_ in modulating food intake centrally.

To gain insights into the role of GPR75 in the central regulation of feeding, we performed transcriptomics analysis (RNA-Seq) of the hypothalamus from WT and *Gpr75^–/–^* mice on a chow diet or on a 2-week HFD. For a FDR below 0.05, only a total of 8 genes were significantly changed in the hypothalamus of chow diet–fed *Gpr75^–/–^* mice, including 5 genes with increased expression and 3 genes with decreased expression (Figure 4, G and H). While on a HFD, a total of 30 genes were significantly changed in the hypothalamus of *Gpr75^–/–^* mice for a FDR below 0.05, including 5 genes with increased expression and 25 genes with decreased expression (Figure 4, G and I). It is worth noting that only 1 gene, proteolipid protein (myelin) 1 (*Plp1*), was markedly decreased in both chow diet– and HFD-fed *Gpr75^–/–^* mice. The major transcriptome change observed only in HFD-fed *Gpr75^–/–^* mice is consistent with the appearance of a strong lean phenotype of *Gpr75^–/–^* mice driven by the HFD. To facilitate interpretation and identify relevant signaling pathways associated with loss of GPR75 in the hypothalamus, we next performed overrepresentation analyses mapping significant genes to the Reactome and Wikipathways databases included in the ConsensusPathDB (18) (Figure 4, J and K). We found an overrepresentation of pathways with important roles in the regulation of energy homeostasis, including TGF-β signaling (19, 20) and signaling by receptor tyrosine kinases (21), and development and function of neurons, including extracellular matrix organization (22), signaling by neurotrophic tyrosine receptor kinases (NTRKs) (23), and glial cell differentiation. These data suggest that GPR75 regulated various signaling pathways after HFD feeding.

### GPR75 is localized in the primary cilia.

**Figure 5:**
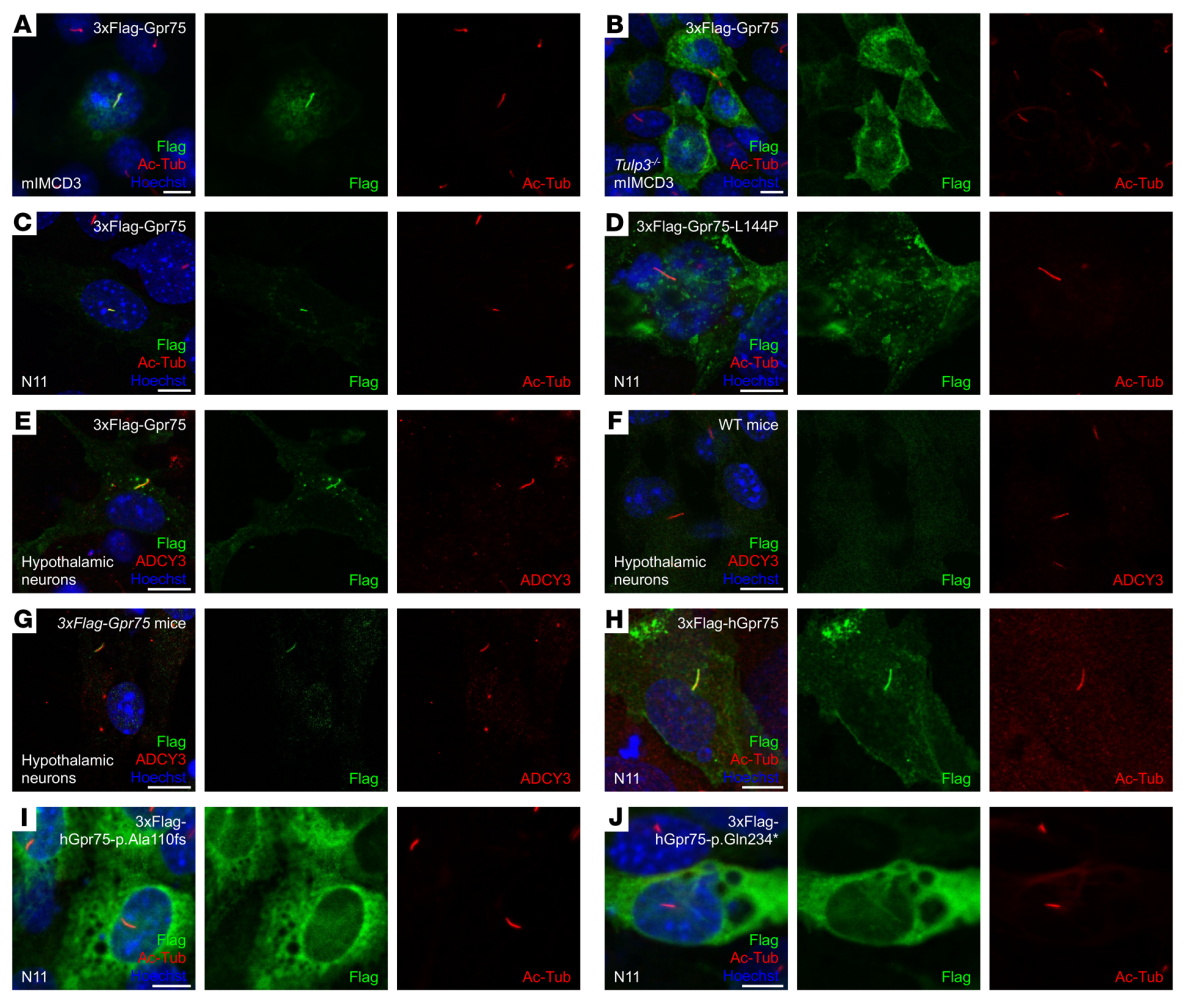
GPR75 is located in the primary cilia. (**A**) mIMCD3 cells expressing 3xFlag-tagged Gpr75 were immunostained with Flag antibody (green), Ac-tubulin (Ac-Tub) (red), and Hoechst 33342 (blue). (**B**) *Tulp3^–/–^* mIMCD3 cells expressing 3xFlag-tagged Gpr75 were immunostained with Flag antibody (green), Ac-tubulin (red), and Hoechst 33342 (blue). (**C** and **D**) N11 cells expressing 3xFlag-tagged Gpr75 WT (**C**) or L144P (**D**) were immunostained with Flag antibody (green), Ac-tubulin (red), and Hoechst 33342 (blue). (**E**) Mouse primary hypothalamic neurons expressing 3xFlag-tagged Gpr75 were immunostained with Flag antibody (green), ADCY3 (red), and Hoechst 33342 (blue). (**F** and **G**) Primary hypothalamic neurons isolated from WT mice (**F**) or homozygous *3xFlag-Gpr75*–knockin mice (**G**) were immunostained with Flag antibody (green), ADCY3 (red), and Hoechst 33342 (blue). (**H**–**J**) N11 cells expressing 3xFlag-tagged human GPR75 WT (**H**), p.Ala110fs (**I**), or p.Gln234* (**J**) were immunostained with Flag antibody (green), Ac-tubulin (red), and Hoechst 33342 (blue). Data are representative of 3 independent experiments. Scale bars: 10 μm.

Primary cilia are present in various cell types and play a crucial role in cell signaling. Dysregulation of cilia or ciliary proteins is closely linked to obesity (24). Many GPCRs are cilia-associated proteins, and their functions within the cilia are essential for regulating food intake and energy expenditure (25, 26). To explore whether GPR75 is a cilia-associated protein, we overexpressed 3xFlag-Gpr75 in mouse inner medullary collecting duct (mIMCD) 3 cells and observed its subcellular localization. Immunofluorescence staining results clearly indicated that the GPR75 protein was localized in the cilia (Figure 5A). TUB-like protein 3 (TULP3) is known to mediate the trafficking of GPCRs into the primary cilia (27, 28). Indeed, GPR75 interacted with TULP3 when expressed in 293T cells (Supplemental Figure 6). In *Tulp3*-knockout (*Tulp3^–/–^*) mIMCD3 cells, GPR75 protein failed to localize in the cilia, suggesting that the ciliary localization of GPR75 was dependent on TULP3 (Figure 5B). Considering the specific expression of GPR75 protein in the brain, we thought it would be interesting to check the ciliary localization of GPR75 in brain neuron–derived cell lines. Therefore, we used the mouse embryonic hypothalamic cell line N11 to examine the subcellular localization of GPR75. Similar to mIMCD3 cells, we found that GPR75 was exclusively localized in the cilia of N11 cells (Figure 5C). However, the L144P-mutant form of GPR75 identified in *Thinner* mice failed to localize in the cilia (Figure 5D), suggesting that ciliary localization is important for the role of GPR75 in energy homeostasis. Besides cell lines, we found that overexpressed GPR75 was also localized in the cilia of mouse primary hypothalamic neurons (Figure 5E). To assess the subcellular localization of endogenous GPR75 protein, we isolated primary hypothalamic neurons from WT control and *3xFlag-Gpr75*–knockin mice. As shown in Figure 5, F and G, endogenous GPR75 protein colocalized with the cilia marker ADCY3, confirming its presence in the cilia. While human GPR75 protein is reported to be expressed on the cell surface and localize in the plasma membrane in human embryonic kidney (HEK) 293 cells, 2 loss-of-function mutations of GPR75 (p.Ala110fs and p.Gln234*) were unable to localize in the plasma membrane (14). However, whether human GPR75 is localized in the primary cilia remains unknown. Similar to mouse GPR75, we observed that human GPR75 was specifically localized in the cilia of N11 cells (Figure 5H). Additionally, 2 mutations of human GPR75 (p.Ala110fs and p.Gln234*) that are associated with lower BMI failed to localize in the cilia (Figure 5, I and J). In conclusion, we found that GPR75 was a cilia-associated protein whose localization in the primary cilia was crucial for its function in regulating body weight in both mice and humans.

### Loss of GPR75 has no effect on the development of obesity in Lep^ob^-mutant mice or Adcy3-mutant mice.

**Figure 6:**
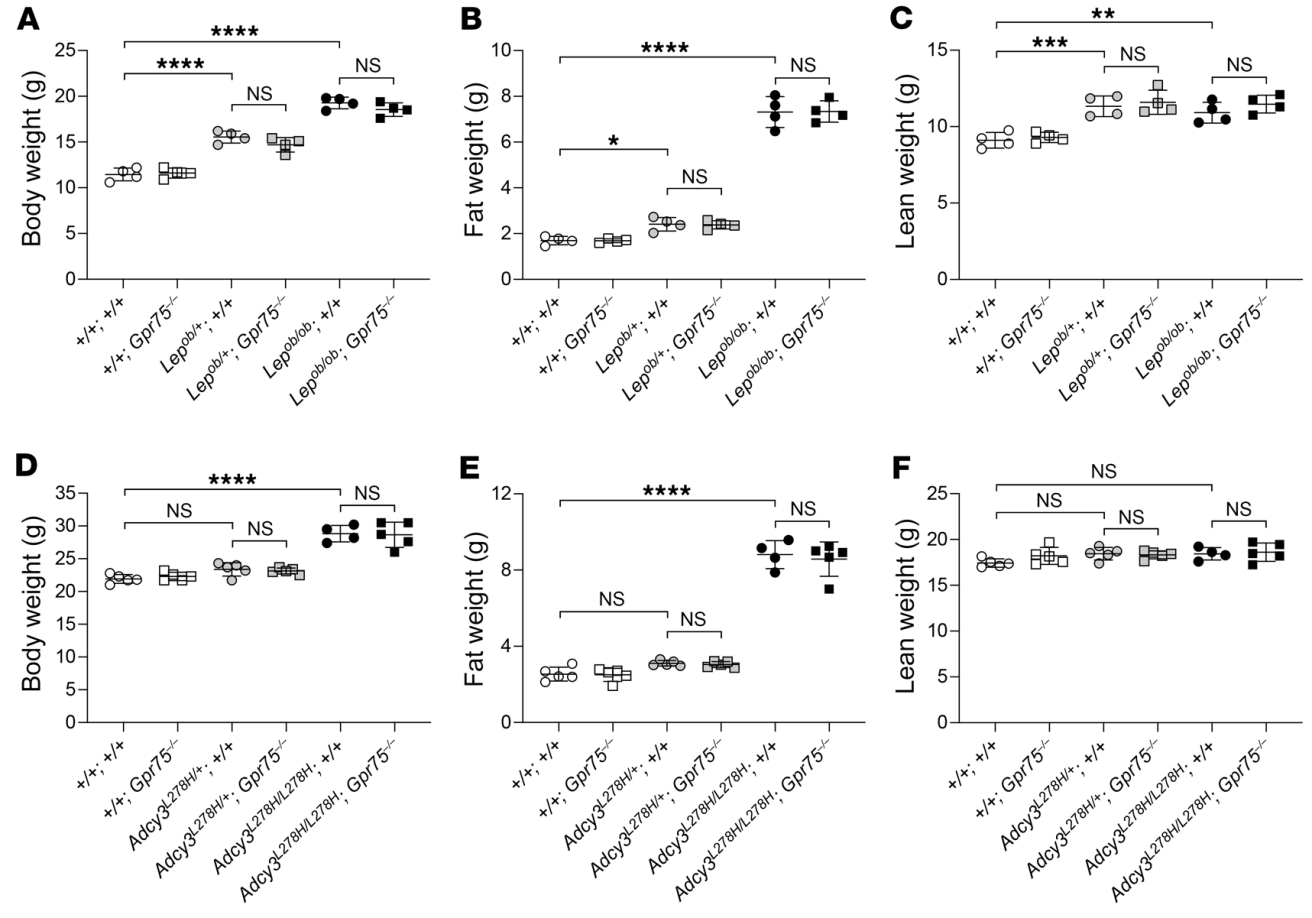
*Gpr75^–/–^* does not inhibit the development of obesity in *Lep^ob^-* or*Adcy3*-mutant mice. (**A**–**C**) Body weight (**A**), fat weight (**B**), and lean weight (**C**) of 5-week-old male mice. (**D**–**F**) Body weight (**D**), fat weight (**E**), and lean weight (**F**) of 8-week-old male mice. Data are presented as the mean ± SD. *P* values were determined by 1-way ANOVA with Holm-Šidák’s multiple-comparison test (**A**–**F**). *P ≤ 0.05, **P ≤ 0.01, ***P ≤ 0.001, and ****P ≤ 0.0001; NS, *P* > 0.05. Data points represent individual mice (**A**–**F**), and data are representative of 3 independent experiments.

Leptin signaling in the brain plays an important role in the regulation of food intake (29). We crossed *Gpr75^–/–^* mice with *Lep^ob^*-mutant mice to determine whether GPR75 is involved in leptin signaling and whether loss of GPR75 would attenuate the obesity phenotype of *Lep^ob^*-mutant mice. As expected, *Lep^ob/+^* and *Lep^ob/ob^* mice had a greater increase in body weight, fat weight, and lean weight than did WT mice at 4 weeks of age (Figure 6, A–C). Complete knockout of *Gpr75* did not reduce the increased fat and lean weight in either *Lep^ob/+^* or *Lep^ob/ob^* mice (Figure 6, A–C). ADCY3 catalyzes the synthesis of cAMP, and its functions within the primary neuronal cilia are essential in regulating body weight (30–32). To assess the genetic interaction between GPR75 and ADCY3, we had to cross *Gpr75^–/–^* mice with *Adcy3^–/–^* mice. Unfortunately, *Adcy3^–/–^* mice are known to be anosmic and have a very high fatality rate within 48 hours of birth (33). During our genetic screening, we identified a viable hypomorphic *Adcy3*-mutant mouse (*Adcy3^L278H/L278H^*) with massive obesity. As shown in Figure 6, D–F, *Adcy3^L278H/L278H^* mice had increased body and fat weights compared with WT mice at 8 weeks of age. Loss of *Gpr75* failed to reduce the development of obesity in *Adcy3^L278H/L278H^* mice (Figure 6, D–F). Taken together, these findings demonstrate that loss of *Gpr75* did not attenuate the obesity phenotype of *Lep^ob^*- or *Adcy3*-mutant mice.

### Testing the ligands of GPR75.

**Figure 7:**
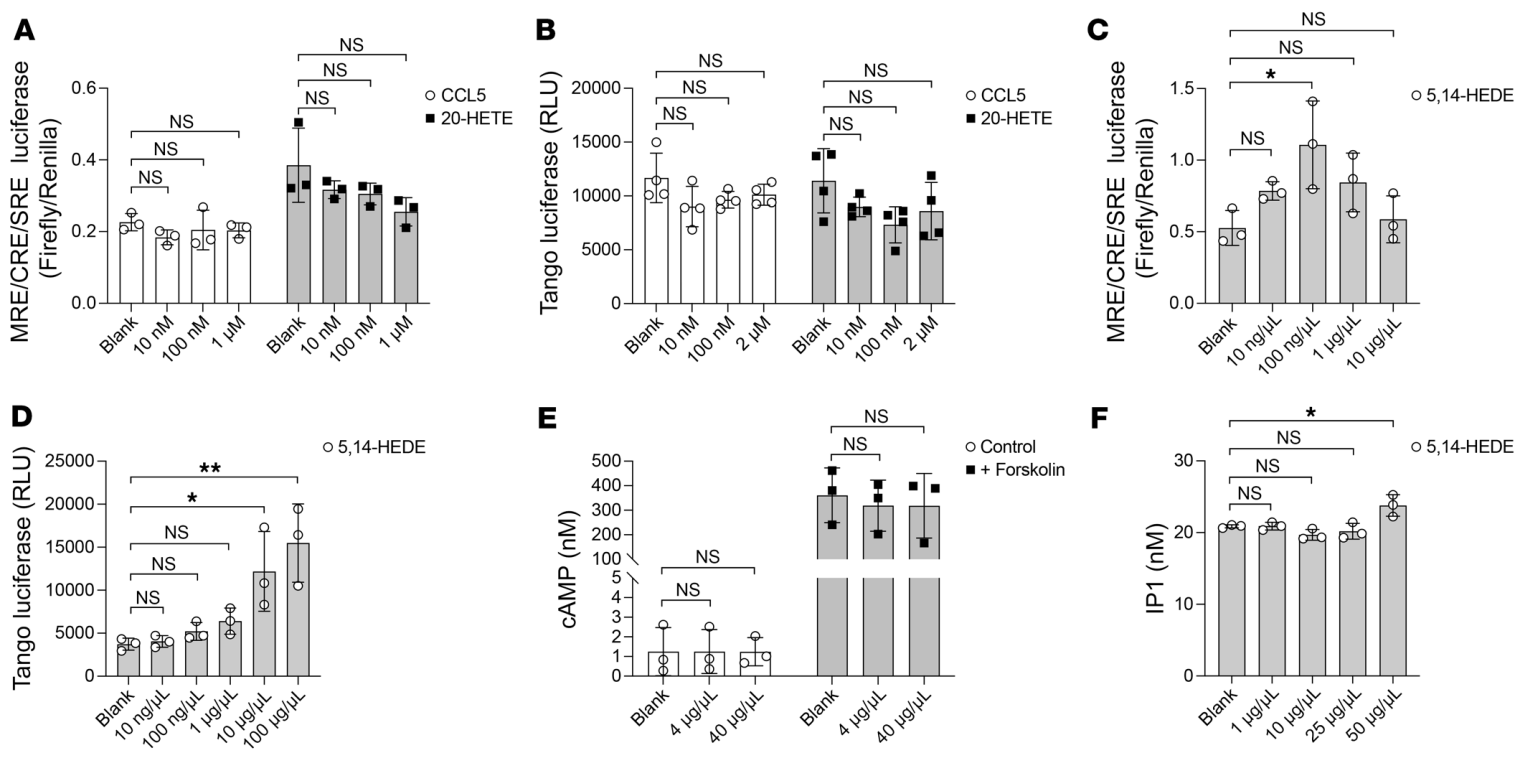
Exploring ligands of GPR75. (**A**) MRE/CRE/SRE luciferase assay. 293T cells expressing human GPR75 and pGL3-MRE/CRE/SRE-luciferase plasmids were treated with different concentrations of CCL5 and 20-HETE. (**B**) PRESTO-Tango β-arrestin recruitment assay in HTLA cells overexpressing GPR75-Tango constructs treated with different concentrations of CCL5 and 20-HETE. (**C**) MRE/CRE/SRE luciferase assay. 293T cells expressing human GPR75 and pGL3-MRE/CRE/SRE-luciferase plasmids were treated with different concentrations of 5,14-HEDE. (**D**) PRESTO-Tango β-arrestin recruitment assay in HTLA cells overexpressing GPR75-Tango constructs treated with different concentrations of 5,14-HEDE. (**E**) cAMP assay. 293T cells expressing human GPR75 were treated with different concentrations of 5,14-HEDE and forskolin. (**F**) IP1 assay. HTLA cells expressing human GPR75 were treated with different concentrations of 5,14-HEDE. Data are presented as the mean ± SD. *P* values were determined by 1-way ANOVA with Holm-Šidák’s multiple-comparison test (**A**–**F**). *P ≤ 0.05 and **P ≤ 0.01; NS, *P* > 0.05. Data points represent individual wells (**A**–**F**), and data are representative of 3 independent experiments.

At present, CCL5 and 20-HETE, are reported to be ligands of GPR75 (15, 16). We conducted 2 distinct assays to test the effect of CCL5 and 20-HETE on GPR75. Initially, we generated a luciferase report construct that contained a multiple response element (MRE), a cAMP response element (CRE), a serum response element (SRE), and a luciferase gene. The MRE/CRE/SRE luciferase assay is capable of detecting agonist effects from Gi-, Gs-, and Gq-coupled receptors as well as the activities of most GPCRs (34, 35). Human GPR75 and the luciferase reporter construct were cotransfected in 293T cells to assess the luciferase activity with different concentrations of these ligands. The second assay we used was PRESTO-Tango assay, which is designed to identify ligands through the G protein–independent β-arrestin recruitment pathway (36). However, we did not observe strong activation of human GPR75 by CCL5 or 20-HETE, whether in the MRE/CRE/SRE luciferase assay or the PRESTO-Tango assay (Figure 7, A and B). Since 20-HETE is prone to oxidation, we used a stable synthetic analog, sodium 20-hydroxyeicosa-5(Z),14(Z)-dienoate (5,14-HEDE), to explore its potency in activating GPR75. In the MRE/CRE/SRE luciferase assay, we observed a modest induction of GPR75 with concentrations exceeding 1 μg/mL 5,14-HEDE (Figure 7C), whereas in the PRESTO-Tango assay, only a high concentration (≥1 μg/mL) of 5,14-HEDE appeared to activate GPR75 (Figure 7D). However, 5,14-HEDE did not increase intracellular cAMP level via GPR75 (Figure 7E), and a very high concentration (50 μg/mL) of 5,14-HEDE seemed to increase intracellular levels of inositol phosphate 1 (IP1) (Figure 7F). These findings do not conclusively establish CCL5, 20-HETE, and 5,14-HEDE as definitive ligands of GPR75. The pursuit of novel GPR75 ligands is worthwhile, particularly those that could potentially regulate food intake.

## Discussion

To identify new regulators of obesity, we used ENU-based phenotype-driven forward genetic screening to identify mutations that change body weight in mice. A missense allele of *Gpr75*, named *Thinner*, was detected in this screen. *Gpr75^–/–^* mice exhibited decreased body fat under both a HFD and a chow diet. Apart from food intake, there were no differences in energy expenditure, physical activity, or food digestion and absorption between WT and *Gpr75^–/–^* mice. Although there are conflicting results regarding the contribution of food intake and energy expenditure in the development of the lean phenotype in GPR75-deficient mice (37, 38), our pair-feeding experiment clearly showed that decreased food intake mainly caused the lean phenotype in *Gpr75^–/–^* mice.

Protein-truncating variants in GPR75 have been linked to lower BMI, and *Gpr75^–/–^* mice have shown resistance to HFD-induced obesity (14). Our study revealed that *Gpr75^–/–^* mice had reduced food intake, confirming the decreased food intake as the primary cause of the observed lean phenotype. However, the mechanism through which GPR75 regulates food intake remains unknown. Utilizing Flag tag–knockin mice, we were able to detect endogenous GPR75 protein levels. The protein’s notably higher expression in the brain compared with other tissues suggests its involvement in regulating energy homeostasis through the central nervous system. Simultaneously, the uniform expression of GPR75 protein across various brain regions makes it challenging to pinpoint the specific brain area(s) in which GPR75 works to regulate body weight. Additionally, it has been reported that GPR75 plays a role in regulating hippocampal activity, and *Gpr75*-knockout mice display altered contextual memory and anxiety-like behaviors (39). Further studies involving specific knockout of *Gpr75* in distinct brain regions could provide insights into its role in obesity and anxiety.

Despite reports identifying 20-HETE and CCL5 as potential ligands for GPR75 (15, 16), our experimental results failed to validate these findings in our system. We did observe modest activation of GPR75 with high concentrations of 5,14-HEDE; however, high concentrations of the compound might also affect normal cellular activities, thereby influencing experimental results. Previous studies have suggested that 20-HETE impairs insulin signaling through GPR75 activation and contributes to the development of insulin resistance (9, 40). In addition, several studies also indicated that deficiency in GPR75 protects against the onset of insulin resistance (14, 37, 38). All mice used in these studies had been on a HFD for longer than 14 weeks, while our data showed that the glucose and insulin metabolism in *Gpr75^–/–^* mice on a HFD for 8 weeks had no abnormalities. This suggests that the improved insulin sensitivity observed in *Gpr75^–/–^* mice under prolonged HFD feeding was a secondary effect of the reduced adiposity and that the resistance of *Gpr75^–/–^* mice to HFD-induced obesity was not due to enhanced insulin sensitivity but rather decreased food intake. Thus, 20-HETE, at least, does not seem to act as a ligand involved in the activation of GPR75 to regulate food intake and body weight. Further studies are essential to identify the specific ligand for GPR75 in the context of obesity.

Primary cilia, particularly in neuronal cells, play an essential role in the regulation of energy homeostasis (24). Mutations in many cilia-associated proteins lead to obesity. Surprisingly, both human and mouse GPR75 were found to be situated in primary cilia, marking GPR75 as the first identified cilia-associated protein whose loss-of-function mutations do not induce obesity but instead lead to leanness in both mice and humans. The presence and function of GPR75 suggest that abnormalities in ciliary signaling may not always lead to obesity. Disruption of distinct cilia signaling pathways may affect energy balance in both directions. In contrast to the obesity phenotype associated with many cilia-associated proteins, the leanness observed in *Gpr75^–/–^* mice was relatively weak under a chow diet. It took approximately 16 weeks for *Gpr75^–/–^* mice to show a reduction in body fat, whereas obese mice typically have increased body fat accumulation within 4–8 weeks. It is plausible that additional “weak” cilia signals capable of decreasing food intake, enhancing energy expenditure, and promoting a leaner state might exist but are currently overlooked and concealed. This phenomenon also suggests that organisms exercise a more cautious and precise regulation of reduced food intake.

The appearance of the lean phenotype of *Gpr75^–/–^* mice requires HFD feeding, as the lean phenotype is very weak on a regular chow diet. Even on a chow diet, *Lep^ob^*- and *Adcy3*-mutant (mice are known to accumulate massive amounts of fat by 6–8 weeks of age, largely due to increased food intake. However, in the present study, the loss of GPR75 (*Gpr75^–/–^*) failed to inhibit the development of obesity in these chow diet–fed *Lep^ob^*- and *Adcy3*-mutant mice. These data suggest that GPR75 does not function downstream of leptin signaling or, alternatively, that GPR75 operates in a novel pathway that runs parallel to the classic leptin/melanocortin signaling to regulate appetite. Another intriguing hypothesis is that GPR75 may somehow “sense” the HFD and activate to increase feeding, serving as an evolutionarily conserved mechanism for our ancestors to store more animal fats when they are available. It would be interesting to study the potential role of GPR75 as a sensor of fatty foods, which might help to explain why humans prefer consuming fatty foods and consequently become obese.

In summary, the close association of GPR75 deficiency with obesity and food intake in both mice and humans makes it a promising drug target for treating obesity. The localization of GPR75 in primary cilia may help us better understand the function of cilia in obesity and eating behavior in the future.

## Methods

### Sex as a biological variable.

Only male animals were used for the CRISPR-knockout studies, with the rationale that male mice are more susceptible to diet-induced obesity. However, both male and female animals were examined in the genetic screening, and similar findings were reported for both sexes. Thus, the findings are expected to be relevant for both sexes, and sex was not considered as a biological variable.

### Mice.

*C57BL/6J* mice (stock 000664) and the *ob* strain (*B6.Cg-Lepob/J*, stock 000632) were purchased from The Jackson Laboratory. The *Thinner* strain (*C57BL/6J-Gpr75^Thinner^*) and *Adcy3^L278H/L278H^* mice were generated by ENU mutagenesis and are described at Mutagenetix (http://mutagenetix.utsouthwestern.edu). *Gpr75*-knockout (*Gpr75^–/–^*) mice and 3xFlag tag Gpr75-knockin (*3xFlag-Gpr75*) mice were generated in our laboratory using the CRISPR/Cas9 system as described previously (41), with *Gpr75* (5′-ATTGGGGACATTCTGAAGCG-3′) small base-pairing guide RNA (sgRNA) and the oligo template with the 3xFlag tag and flanking sequence: 5′-TGAGCTGAGATCCTGACTCTTTTCCTGCTGAATTTATTTTTTTGAGAACACAAGAAAGAGACACCTCTCTCTGAAGatggactacaaagaccatgacggtgattataaagat catgatatcgattacaaggatgacgatgacaagATGAACACAAGTGCCCCGCTTCAGAATGTCCCCAATGCCACCTTGCTAAACATGC-3′. The sex and age of each mouse used in the experiment are specified in the corresponding figure legends and experiment descriptions. Littermate controls were used throughout the study and are clearly mentioned in the results and figure legends. All mice were fed a standard chow diet (2016 Teklad Global 16% Protein Rodent Diet) except mice with diet-induced obesity, which were fed a HFD (60 kcal% fat, D12492, Research Diets) or an unsaturated HFD (45 kcal% fat [Mostly safflower oil], D05122101, Research Diets) from 6 weeks of age. All mice were housed at room temperature (23°C) unless otherwise indicated. Mice were observed daily to ensure good health status and were maintained at The University of Texas Southwestern Medical Center.

### Plasmids.

PCR was carried out using mouse brain cDNA as the template and oligonucleotide primers designed to obtain the coding DNA sequence (CDS) of the mouse *Gpr75* (NM_175490.4) and *Gnaq* (NM_008139.6). Human HeLa cell cDNA was used to obtain the CDS of the human *GPR75* (NM_006794.4). These genes were cloned into the HA-tagged or 3xFlag-tagged pCMV vector for transient expression. The mouse *Gpr75* and human *GPR75* mutants were generated with PCR mutagenesis. The pGL3-MRE/CRE/SRE-luciferase vector was constructed as previously reported (35). The MRE/CRE/SRE fragment was artificially synthesized and inserted into the pGL3-basic vector. The GPR75-Tango plasmid was a gift from Bryan Roth (Addgene plasmid 66372; http://n2t.net/addgene:66372; RRID: Addgene_66372) (36). All constructs were verified by sequencing.

### Cell culture and transfection.

The 293T cells were purchased from American Type Culture Collection (ATCC). The N11 cells were obtained from Xiaoyong Yang at Yale University (New Haven, Connecticut, USA). The mIMCD3 WT and *Tulp3^–/–^* cells were obtained from Saikat Mukhopadhyay at the University of Texas Southwestern Medical Center (Dallas, Texas, USA). The HTLA cells (HEK293-derived cells containing stable integrations of a tTA-dependent luciferase reporter and β-arrestin2-TEV fusion gene) were obtained from Gilad Barnea at Brown University (Providence, Rhode Island, USA). These cells were grown in culture medium (DMEM [Gibco, Thermo Fisher Scientific], 10% vol/vol FBS [ATCC], and 1% penicillin-streptomycin antibiotics [Gibco]) at 37°C with 5% CO_2_. Transfection of plasmids was carried out using Lipofectamine 2000 (Life Technologies, Thermo Fisher Scientific) according to the manufacturer’s instructions. Cells were harvested between 36 and 48 hours of transfection.

### Isolation of primary hypothalamic neurons.

Primary hypothalamic neurons were isolated following a standard protocol (42). First, culture dishes are coated with poly-d-lysine to prepare for cell attachment. Surgical tools were sterilized and kept in ethanol, and wash media (DMEM [Gibco], 1% penicillin-streptomycin antibiotics [Gibco]) were prepared and kept on ice. Pregnant mice (16–19 days) were euthanized, and embryos were collected. The brains were isolated from the embryos, with careful dissection to avoid damage and contamination. The hypothalamus was specifically identified and isolated, rinsed and washed with PBS, and digested with papain-trypsin buffer (2 mg/mL papain [MilliporeSigma] plus 0.05% trypsin-EDTA [Gibco] in wash medium). After neutralizing the enzyme activity, tissues were dissociated into single cells, and the cell density was calculated. Isolated cells were seeded in the coated culture plates with plating medium (Neurobasal medium [Gibco], 10% vol/vol FBS [ATCC], 1% penicillin-streptomycin antibiotics [Gibco], 2 mM l-glutamate [Gibco]) and allowed to attach. Finally, the neurons were maintained in complete culture medium (Neurobasal medium [Gibco], 1% N2 supplement [Gibco], 2% B27 supplement [Gibco], 2 mM l-glutamate [Gibco], 1% penicillin-streptomycin antibiotics [Gibco]). The medium was refreshed by replacing half of it every 3–4 days.

### Metabolic analysis of mice.

The mice underwent a 6-hour fasting period (from 7 am to 1 pm) for both the GTT and ITT. Blood glucose levels were assessed using the AlphaTRAK glucometer and test strips. Following the initial blood glucose measurement, the GTT was initiated by an i.p. injection of a 10% glucose solution (1 g/kg; MilliporeSigma), and blood glucose levels were monitored at set time points over the next 2 hours. The ITT was initiated by an i.p. injection of human insulin (0.75 U/kg; MilliporeSigma), and blood glucose levels were measured at designated time points over the subsequent 2 hours. Magnetic resonance spectroscopy (MRS) was conducted on live mice using EchoMRI Body Composition Analyzers with default settings. A cold tolerance test was performed as previously described (43). In brief, internal body temperatures of the mice were tracked by implanting a temperature transponder (IPTT-300, Bio Medic Data Systems) under the skin and recording temperatures with a portable reader (DAS-8007-IUS, Bio Medic Data Systems). To subject the mice to acute cold exposure, they were individually housed in 6°C cold chambers without access to food, and body temperature was assessed at specified time points. Metabolic cage measurements were performed using the TSE PhenoMaster system. Mice were acclimatized for 5 days in metabolic cages before the actual measurement. For the pair-feeding experiment, WT mouse (pair-fed group) was offered the amount of HFD eaten by the *Gpr75^–/–^* mouse (comparison group) on the previous day, beginning at 6 weeks of age.

### Serum chemistries and ELISA.

The mice underwent a 6-hour fasting period (from 7 am to 1 pm) before all blood sample collections. Insulin and leptin levels in the serum were measured using ELISA kits from Crystal Chem, following the manufacturer’s instructions. Triglyceride levels were assessed using the Infinity Triglycerides Liquid Stable Reagent from Thermo Fisher Scientific, with the Matrix Plus Chemistry Reference Kit from Verichem Laboratories as the standard for measurement. Cholesterol levels were determined using the Infinity Cholesterol Liquid Stable Reagent from Thermo Fisher Scientific, with the Matrix Plus Cholesterol Reference Kit from Verichem Laboratories used as the measurement standard.

### Immunohistochemistry and immunostaining.

Tissue samples for routine histology and special stains were obtained from anesthetized mice and fixed following standard protocols, with adjustments made for tissue size and staining requirements. Samples for H&E staining were fixed for 48 hours in 10% (vol/vol) neutral-buffered formalin and stored briefly in 75% (vol/vol) ethanol. These sections were stained for histopathological evaluation by regressive H&E on a Sakura Finetek DRS-601 robotic staining system using Leica SelecTech reagents (hematoxylin 560 and alcoholic eosin Y 515).

For immunostaining, mIMCD3 cells and N11 cells cultured in chambers were washed with PBS, fixed in freshly prepared 4% formaldehyde in PBS buffer for 10 minutes at room temperature, rinsed again with PBS, and then blocked with PBSA (PBS and 3% BSA by weight) for 1 hour. Following this, the cells were incubated with a primary antibody diluted in PBSA overnight at 4°C, washed with PBS, incubated with a secondary antibody diluted in PBSA for 30 minutes at room temperature, and finally mounted using a mounting medium (Life Technologies). The following primary antibodies were used in this study: mouse anti-Flag (M2, MilliporeSigma, 1:500) and rabbit anti–Ac-tubulin (D20G3, Cell Signaling Technology [CST], 1:800). The following secondary antibodies were used in the study: Alexa Fluor 488 Goat Anti–mouse IgG (H + L) (115-545-166, Jackson ImmunoResearch, 1:500) and Rhodamine Red-X Goat Anti–rabbit IgG (H + L) (111-295-144, Jackson ImmunoResearch, 1:500). Hoechst (CST) was used to stain nuclei.

### Sample preparation, immunoprecipitation, and Western blot analysis.

For Western blot analysis, cells were collected in 1×NuPAGE LDS sample buffer (Life Technologies) containing 2.5% 2-mercaptoethanol (MilliporeSigma). For immunoprecipitation, tissues or cells underwent lysis in Nonidet P-40 lysis buffer (50 mM Tris-Cl, pH 8.0, 0.1 M NaCl, 10 mM sodium fluoride, 1 mM sodium vanadate, 1% Nonidet P-40, 10% glycerol, 1.5 mM EDTA, and Protease Inhibitor Mixture) for 30 minutes at 4°C. After centrifugation, lysates were incubated with Flag antibody–conjugated beads for 2 hours at 4°C. The beads were subsequently washed 3 times with 1 mL Nonidet P-40 lysis buffer and eluted using 3xFlag peptides for 30 minutes at 4°C. For immunoprecipitation intended for mass spectrometric analysis, the procedure was similar except for the higher cell count and longer lysis time. Mass spectrometric analysis was carried out following established methods (44). In a standard Western blot, samples were separated using NuPAGE 4%–12% Bis-Tris gels (Thermo Fisher Scientific), transferred onto NC membranes (Bio-Rad), probed with a primary antibody overnight at 4°C, then incubated with the secondary antibody for 1 hour at room temperature, and finally visualized using a chemiluminescent substrate (Thermo Fisher Scientific). The following primary antibodies were used in this study: mouse anti-HA (HA-7, MilliporeSigma, 1:5,000), anti-Flag (M2, MilliporeSigma, 1:5,000), and rabbit anti-GAPDH (D16H11, CST, 1:2,000). The following secondary antibodies were used in this study: goat anti–mouse IgG (H + L) HRP (115-035-146, Jackson ImmunoResearch, 1:5,000) and goat anti–mouse IgG (light-chain specific) HRP (115-035-174, Jackson ImmunoResearch, 1:5,000).

### RNA isolation, reverse transcription, and reverse transcription quantitative PCR.

Tissue samples or cells were lysed in TRIzol (Invitrogen, Thermo Fisher Scientific) for RNA isolation with the PureLink RNA Mini Kit (Invitrogen) following a standard protocol. Reverse transcription was carried out with SuperScript III First-Strand Synthesis SuperMix (Life Technologies) following a standard protocol. Reverse transcription quantitative PCR (RT-qPCR) was conducted using the ABI StepOnePlus system with Powerup SYBR Green Master Mix (Life Technologies). Relative quantification was carried out using the 2^–ΔΔCt^ method. The following primer pairs were used: *Gpr75*, 5′-GGCGATGATGACTCTAGCCC-3′ (forward), 5′-GTGCCAAAGAAGATAAGCCAGC-3′ (reverse); *Polr2a*, 5′-CAAGATGCAAGAGGAGGAAGAG-3′ (forward), 5′-TGTTGTCTGTCTGAGGTAAGTG-3′ (reverse); *Gpr75* number 1, 5′-GATGAACACAAGTGCCCCGC-3′ (forward), 5′-GGCAAGCAGAAAAGTGCAGG-3′ (reverse); *Gpr75* number 2, 5′-CTTCTTTGGCACATGCTCGTC-3′ (forward), 5′-GTGGCATTGGGGACATTCTGAAG-3′ (reverse).

### Inositol phosphate 1 assay.

IP1 accumulation was measured utilizing the IP-One Gq kit (62IPAPEB, Cisbio) following the manufacturer’s instructions. Briefly, HTLA cells were seeded onto 96-well plates (Corning) at 5 × 10^4^ cells/well in 100 μL medium and transfected with pCMV-human-GPR75. Twenty-four hours after the transfection, the HTLA cells were starved in serum-free DMEM for 2 hours. Subsequently, cells were rinsed and exposed to specific reagents and their corresponding controls for 2 hours. Each well received the IP1 d2 reagent (acceptor) followed by the IP1 Tb cryptate antibody (donor), and the plate was sealed and incubated for 1 hour. Standards and samples were read using a SpectraMax iD5 multimode microplate reader (Molecular Devices) with a homogeneous time-resolved fluorescence (HTRF) protocol (Ex 350 nm, Em 665/620 nm HTRF).

### cAMP assay.

293T cells were seeded onto a 24-well plate and transfected with pCMV-human-GPR75. Twenty-four hours after transfection, 293T cells were treated with the appropriate reagents for 2–4 hours. Cells were treated with 0.5 mM IBMX for 30 minutes and were then either left untreated or treated with 1 μM forskolin for 15 minutes. The cAMP concentrations of all wells were assessed with the Cyclic AMP XP Assay Kit (4339S, CST) following the manufacturer’s protocol.

### β-Arrestin recruitment assay.

The PRESTO-Tango system was utilized to screen ligand-receptor activation via the G protein–independent β-arrestin recruitment pathway (36). HTLA cells were plated onto 96-well plates (Corning) at 5 × 10^4^ cells/well in 100 μL medium and transfected with GPR75-Tango for 24 hours. Subsequently, the cells underwent a 2-hour serum-free DMEM starvation period. After starvation, the cells were rinsed and exposed to various reagents, incubating at 37°C with 5% CO_2_ for 2 hours. Following this incubation, the treatment was removed, and fresh complete media (DMEM supplemented with 20% FBS) were replenished, allowing overnight incubation at 37°C with 5% CO_2_. The next day, the plates were retrieved, and luminescence was measured using the Steady-Glo Luciferase Assay System (E2520, Promega) following the standard protocol.

### Luciferase assay.

Cells were seeded in 96-well plates (Corning) at a density of 5 × 10^4^ cells per well in 100 μL medium and left to incubate at 37°C overnight. Ligands at different concentrations were dissolved in 11 μL medium and added to each well. The cells were incubated at 37°C for 6 hours. Then, the luminescence signal was measured immediately using the Steady-Glo Luciferase Assay System (E2520, Promega).

### RNA-Seq.

Total RNA was extracted from tissues with the TRIzol and PureLink RNA Mini Kit (Invitrogen) following a standard protocol. cDNA synthesis and library preparation were carried out using the SMART-Seq mRNA LP kit (Takara, 634768). Sequencing was performed on the Illumina NovaSeq 6000 platform (PE150, raw data >6 GB/sample). Sequencing data were analyzed using Astrocyte, which is a scientific workflow platform developed by BioHPC at UT Southwestern Medical Center (https://portal.biohpc.swmed.edu/content/). Differentially expressed genes were analyzed with DESeq2 using raw counts. Overrepresentation analyses were performed with ConsensusPathDB (http://cpdb.molgen.mpg.de) (18).

### Statistics.

Data are presented as the mean ± SD in all graphs depicting error bars. The statistical significance of differences between experimental groups was determined by 2-tailed, unpaired Student’s *t* test, 1-way ANOVA with Holm-Šidák’s multiple-comparison test, a mixed-effects model with Holm-Šidák’s multiple comparisons test, or a linear correlation with a 2-tailed comparison of slope and intercept using GraphPad Prism 10 (GraphPad Software). A *P* value above 0.05 was considered statistically significant.

### Study approval.

All mouse experiments in this study were approved by the IACUC of the University of Texas Southwestern Medical Center.

### Data availability.

The RNA-Seq data generated in this publication were deposited in the NCBI’s Gene Expression Omnibus (GEO) database (GEO GSE254239). The corresponding author will provide all requested materials, data sets, and protocols, without restriction, upon request. See the Supporting Data Values file for all data shown.

## Author contributions

YJ and ZZ conceptualized the study, designed the methodology, curated the data, performed visualization, conducted formal analysis. ZZ acquired funding. YJ and YX performed experiments. ZZ administered the project. ZZ provided resources and software. ZZ supervised the study. YJ validated the study results. YJ and ZZ wrote the original draft and reviewed and edited the manuscript.

## Supplementary Material

Supplemental data

Unedited blot and gel images

Supporting data values

## Version 1 08/13/2024

In-Press Preview

## Version 2 10/01/2024

Electronic publication
