# Supplementary material for: Central regulation of feeding and body weight by ciliary GPR75
Source: J Clin Invest. 2024 Aug 13;134(19):e182121. doi: 10.1172/JCI182121 (PMC11444156; doi:10.1172/JCI182121)
Supplement: Supplemental data [file jci-134-182121-s139.pdf]

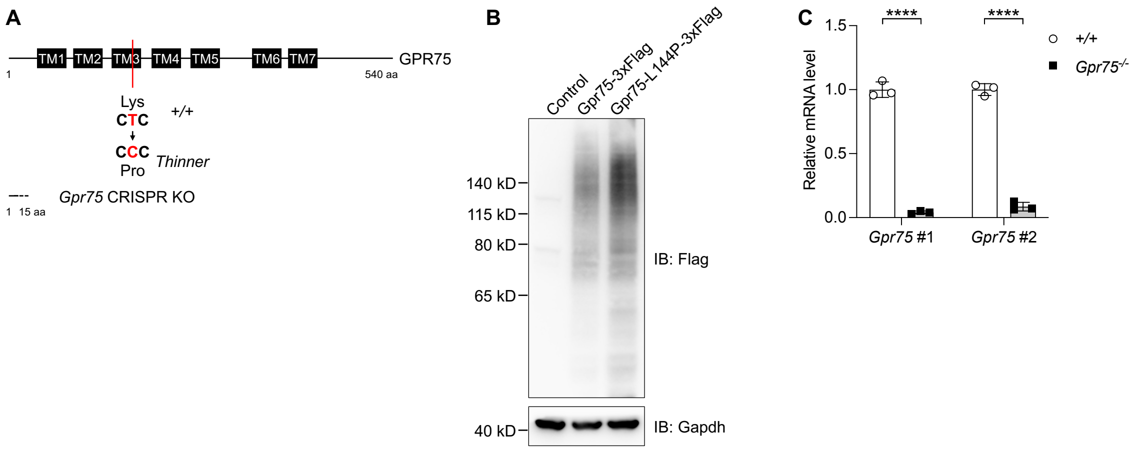

Figure S1. Characterization of the *Thinner* mutation and *Gpr75*$^{-/-}$ mice. (**A**) Protein domain diagrams of mouse GPR75 WT form, with the *Thinner* mutation, and truncated form generated by CRISPR KO. (**B**) Immunoblot analysis of 3xFlag tagged WT and *Thinner* mutant (L144P) GPR75 proteins expressed in 293T cells. Gapdh was used as a loading control. (**C**) Relative mRNA levels of *Gpr75* in the brain lysates of WT and *Gpr75*$^{-/-}$ mice were measured. *Gpr75* #1 and *Gpr75* #2 represent two sets of primers spanning the *Gpr75* KO site used for the qPCR assay. Data are presented as means ± SD. *P* values were determined by two-tailed unpaired Student's *t* test (**C**). *$P \leq 0.05$; **$P \leq 0.01$; ***$P \leq 0.001$; ****$P \leq 0.0001$; ns, not significant with $P > 0.05$. Data points represent individual mice (**C**). Data are representative of two independent experiments.

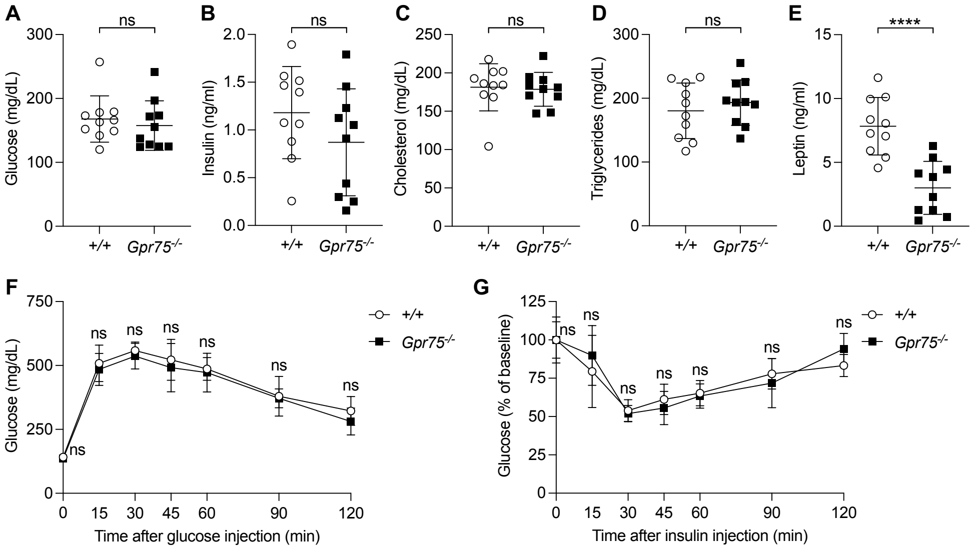

Figure S2. The blood index of *Gpr75*$^{-/-}$ mice. (**A**-**E**) Serum glucose (**A**), insulin (**B**), cholesterol (**C**), triglycerides (**D**), and leptin (**E**) in 14-week-old male mice fed with HFD for 8 weeks after a 6-h fast. (**F**) Glucose tolerance test. Blood glucose was measured at indicated times after i.p. glucose injection in 14-week-old male mice fed with HFD for 8 weeks (n = 5). (**G**) Insulin tolerance test. Blood glucose was measured at indicated times after i.p. insulin injection in 14-week-old male mice fed with HFD for 8 weeks (n = 5). Data are presented as means ± SD. *P* values were determined by two-tailed unpaired Student's *t* test (**A**-**E**) or mixed-effects model with Holm-Sidak's multiple comparisons test (**F**-**G**). *$P \leq 0.05$; **$P \leq 0.01$; ***$P \leq 0.001$; ****$P \leq 0.0001$; ns, not significant with $P > 0.05$. Data points represent individual mice (**A**-**E**). Data are representative of two independent experiments.

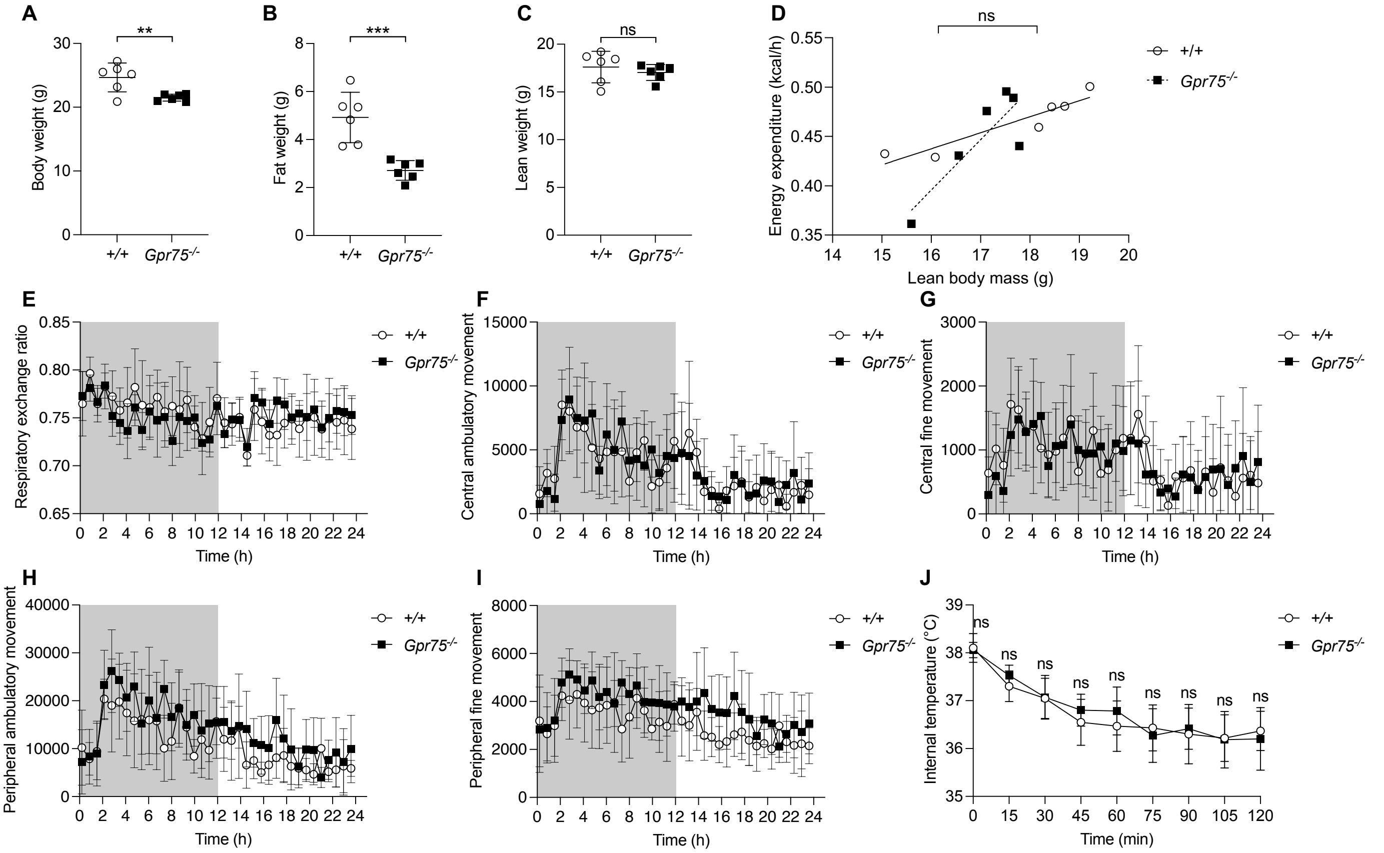

Figure S3. Energy expenditure and physical activity of *Gpr75*$^{-/-}$ mice fed with HFD for 2 weeks. (**A**-**C**) Body weight (**A**), fat weight (**B**), and lean weight (**C**) of 8-week-old male *Gpr75*$^{-/-}$ mice (n = 6) and WT littermates (n = 6) fed with HFD for 2 weeks. These mice were used for metabolic cage experiments in (**D**)-(**I**). (**D**-**I**) Metabolic cage measurements of energy expenditure (**D**), respiratory exchange ratio (**E**), central fine movement (**F**), central ambulatory movement (**G**), peripheral fine movement (**H**), and peripheral ambulatory movement (**I**) of 8-week-old male *Gpr75*$^{-/-}$ mice (n = 6) and WT littermates (n = 6) housed at 23°C. Dark color indicates dark phase when light was off. (**J**) Internal temperature of 10-week-old male mice fed with HFD for 4 weeks housed in the cold in the absence of food (n = 7 WT, 7 *Gpr75*$^{-/-}$). Data are presented as means ± SD. *P* values were determined by two-tailed unpaired Student's *t* test (**A**-**C**) or mixed-effects model with Holm-Sidak's multiple comparisons test (**J**). A linear correlation with a two-tailed comparison of slope and intercept was calculated and compared between different mouse groups (**D**). **P* ≤ 0.05; ***P* ≤ 0.01; ****P* ≤ 0.001; *****P* ≤ 0.0001; ns, not significant with *P* > 0.05. Data points represent individual mice (**A**-**D**). Data are representative of two independent experiments.

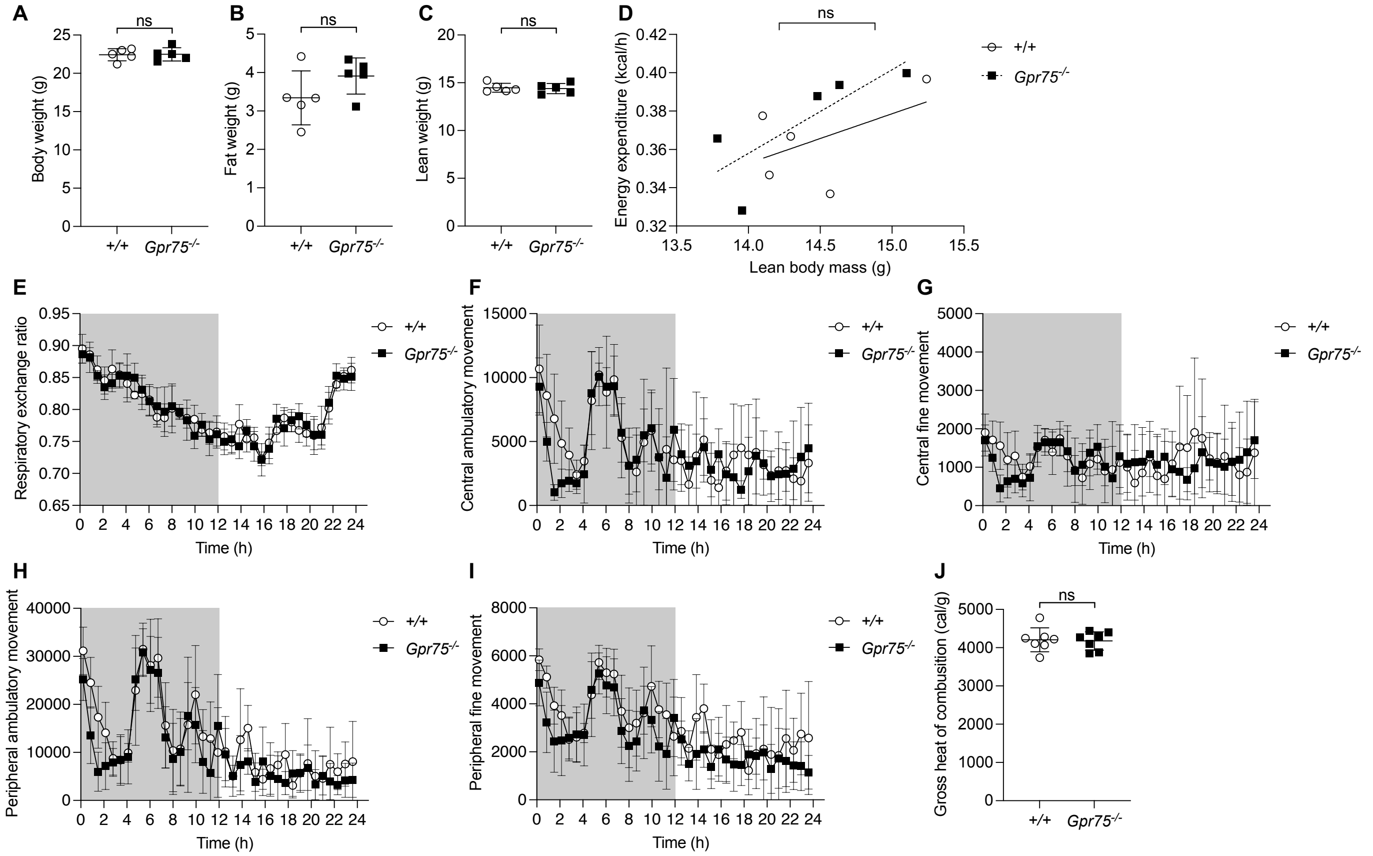

Figure S4. Energy expenditure and physical activity of *Gpr75$^{-/-}$* mice on pair feeding. (**A**-**C**) Body weight (**A**), fat weight (**B**), and lean weight (**C**) of 8-week-old male *Gpr75$^{-/-}$* mice (n = 6) and WT littermates (n = 6) on pair feeding. These mice were used for metabolic cage experiments in (**D**)-(**I**). (**D-I**) Metabolic cage measurements of energy expenditure (**D**), respiratory exchange ratio (E), central fine movement (**F**), central ambulatory movement (**G**), peripheral fine movement (**H**), and peripheral ambulatory movement (**I**) of 8-week-old male *Gpr75$^{-/-}$* mice (n = 6) and WT littermates (n = 6) housed at 23°C. Dark color indicates dark phase when light was off. (**J**) Fecal energy density of 10-week-old male *Gpr75$^{-/-}$* mice and WT littermates fed with HFD. Data are presented as means ± SD. *P* values were determined by two-tailed unpaired Student's *t* test (**A-C**, **J**). A linear correlation with a two-tailed comparison of slope and intercept was calculated and compared between different mouse groups (**D**). **P* ≤ 0.05; ***P* ≤ 0.01; ****P* ≤ 0.001; *****P* ≤ 0.0001; ns, not significant with *P* > 0.05. Data points represent individual mice (**A-D**, **J**). Data are representative of two independent experiments.

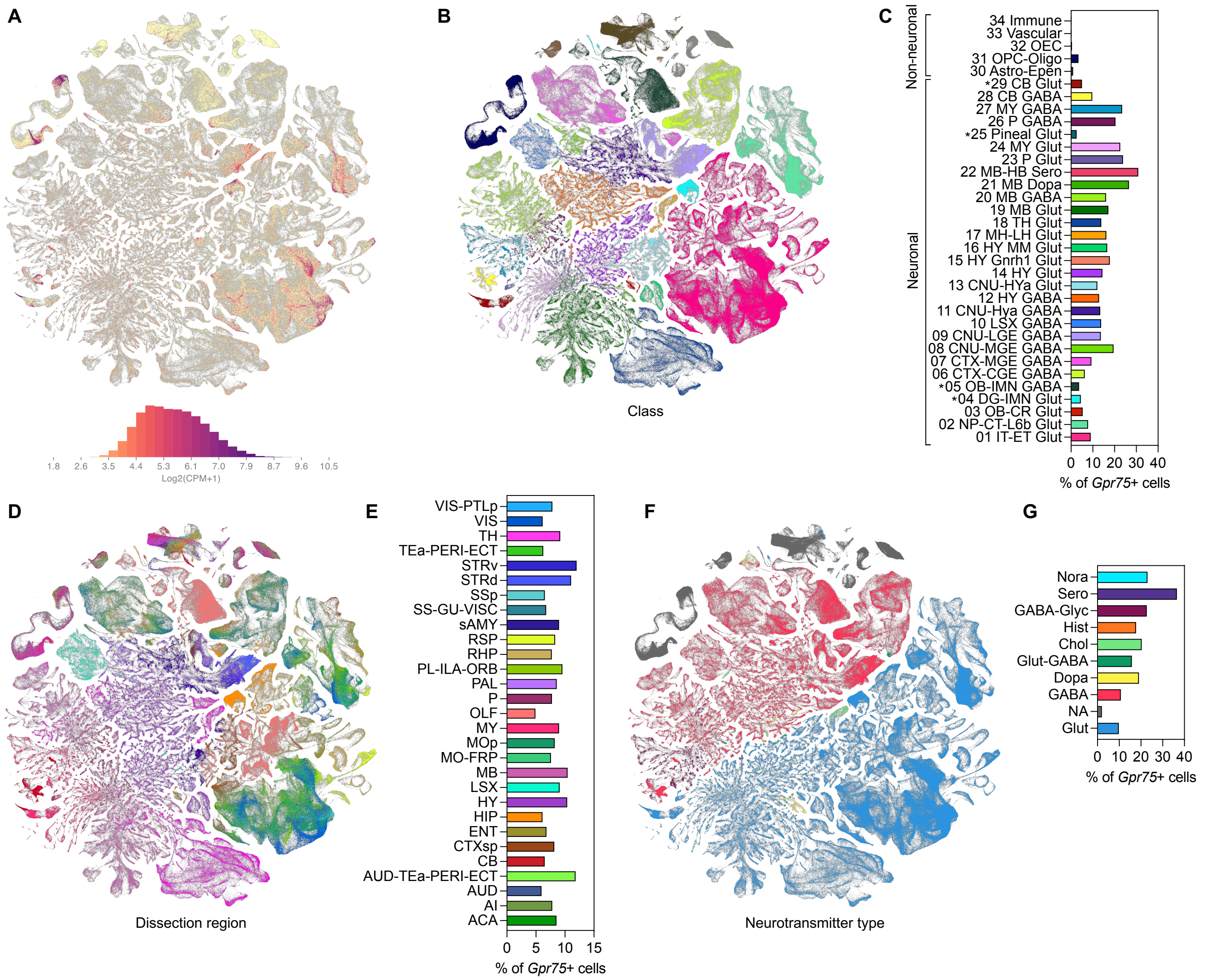

Figure S5. scRNA-seq data analysis of *Gpr75* expression profile in mouse brain. (**A**) UMAP representation of *Gpr75* expression in all brain cells from the Allen Brain Cell Atlas. The bottom plot shows the cell counts (y-axis) versus *Gpr75* expression levels [Log2(CPM+1), x-axis]. (**B**) UMAP representation of all cell types colored by class. (**C**) Quantification of the percentage of *Gpr75*+ cells among the 34 classes. (**D**) UMAP representation of all cell types colored by dissection region. (**E**) Quantification of the percentage of *Gpr75*+ cells among the 29 dissection regions. (**F**) UMAP representation of all cell types colored by major neurotransmitter type. (**G**) Quantification of the percentage of *Gpr75*+ cells among the 10 neurotransmitter types.

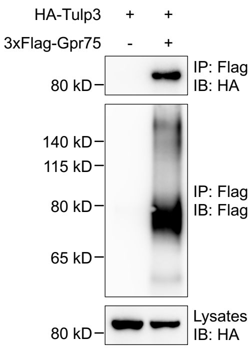

Figure S6. GPR75 interacts with TULP3. Immunoblot analysis of immunoprecipitates (top and middle) or lysates (bottom) of 293T cells expressing HA-tagged Tulp3 and 3xFlag-tagged Gpr75. Data are representative of two independent experiments.
