# Supplementary material for: Central regulation of feeding and body weight by ciliary GPR75
Source: J Clin Invest. 2024 Aug 13;134(19):e182121. doi: 10.1172/JCI182121 (PMC11444156; doi:10.1172/JCI182121)

Unedited blot and gel images

Figure S1B

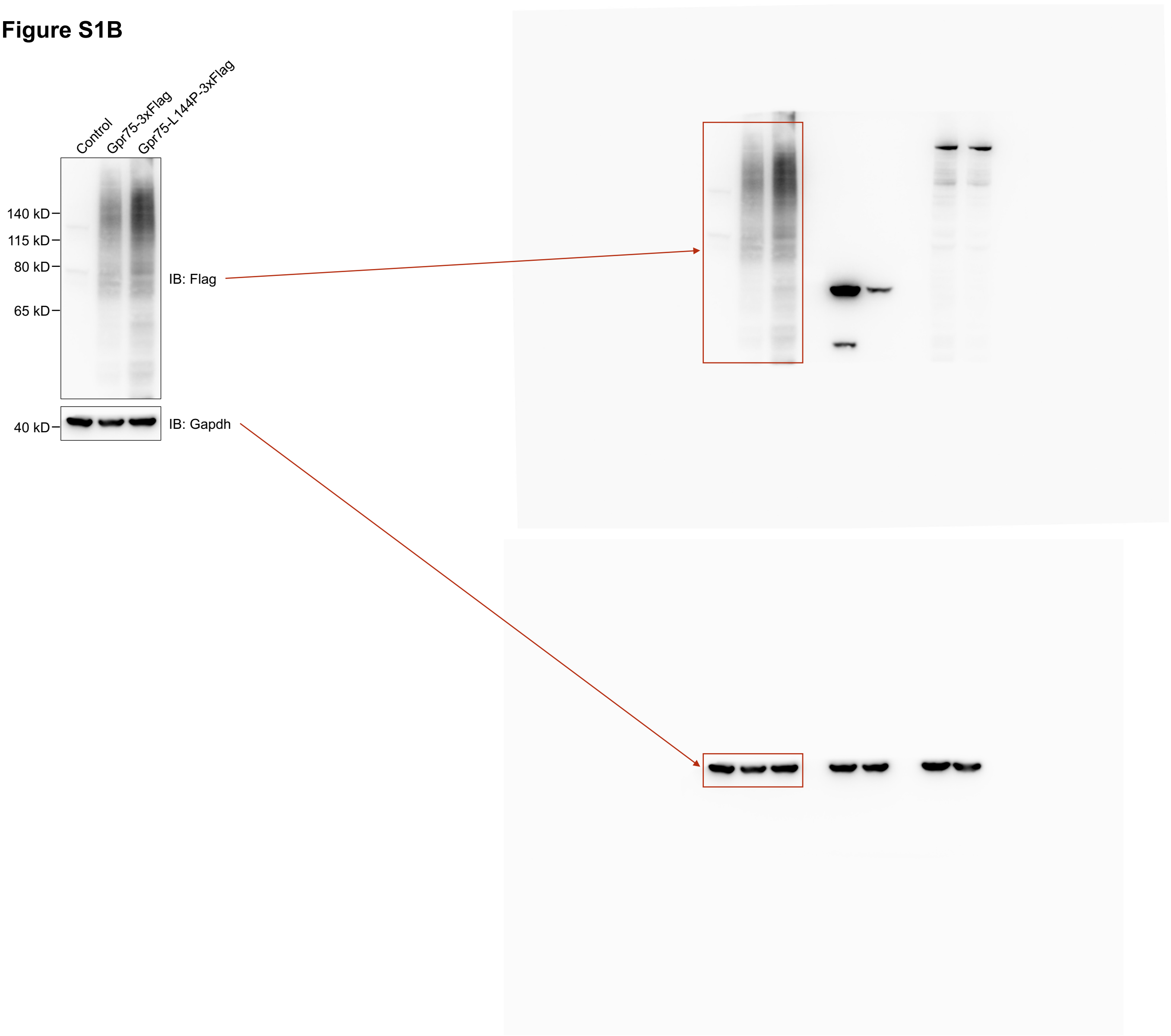

Unedited blot images

Figure 4C

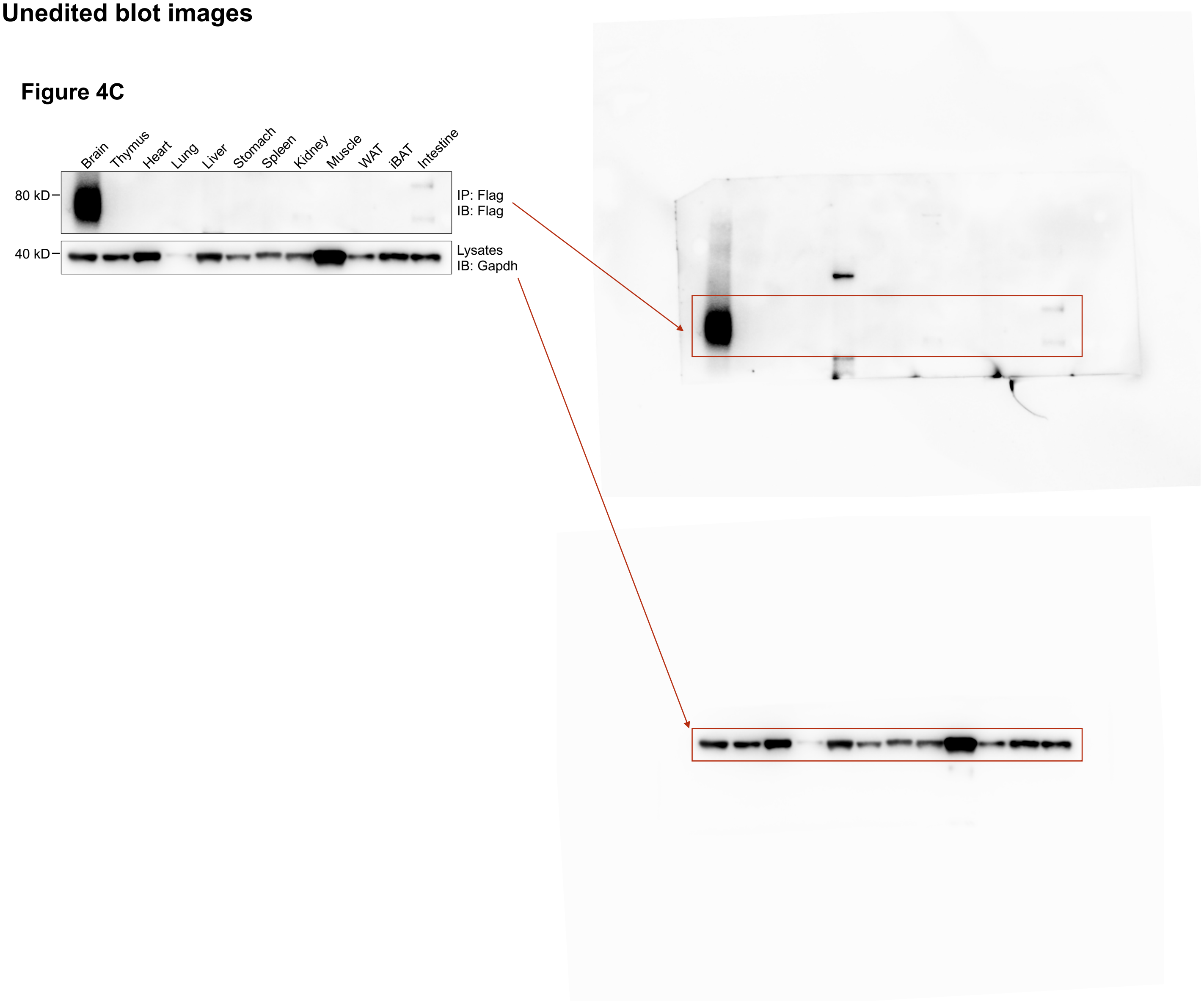

Unedited blot images

Figure 4D

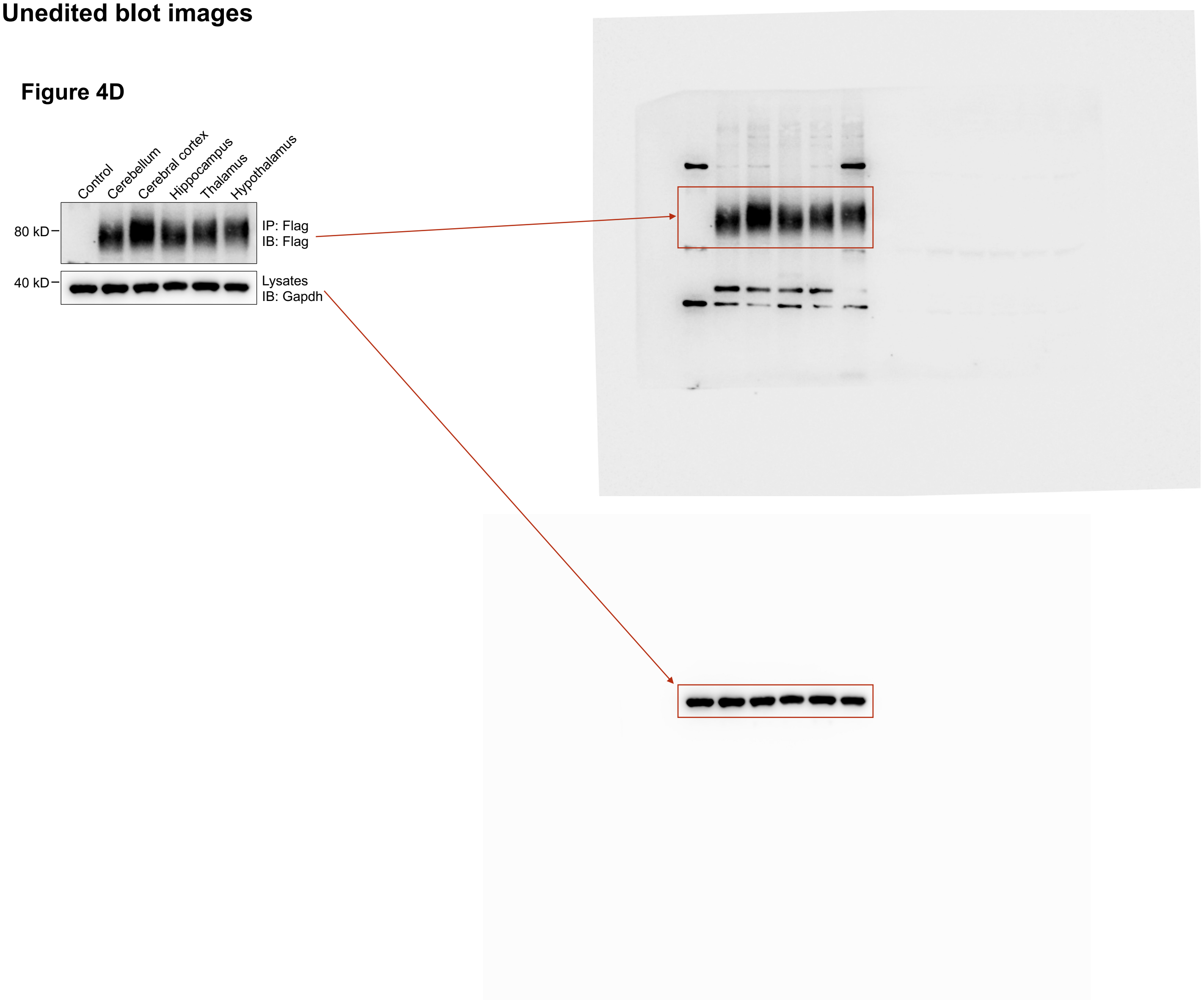

Unedited blot images

Figure 4F

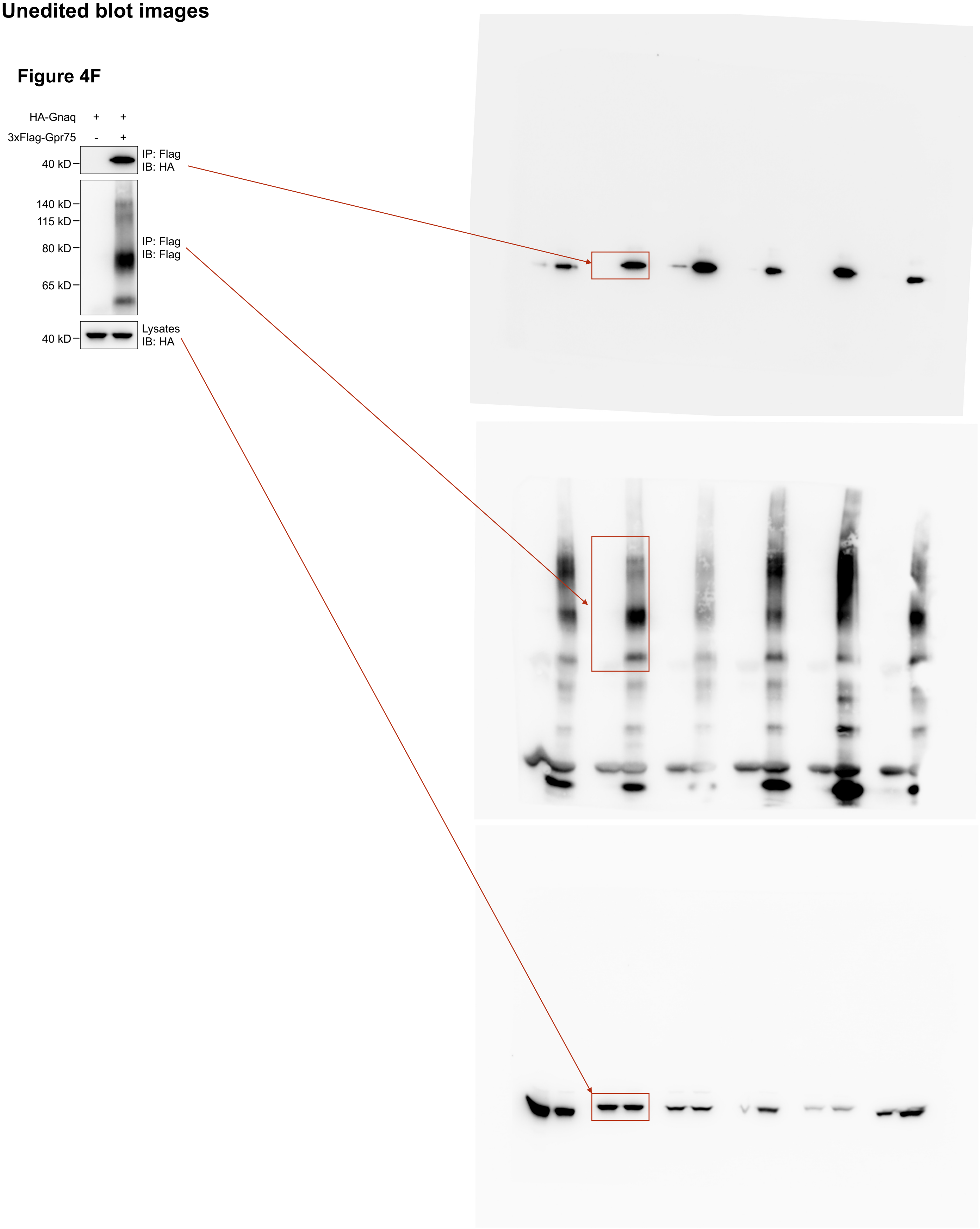

Unedited blot images

Figure S6

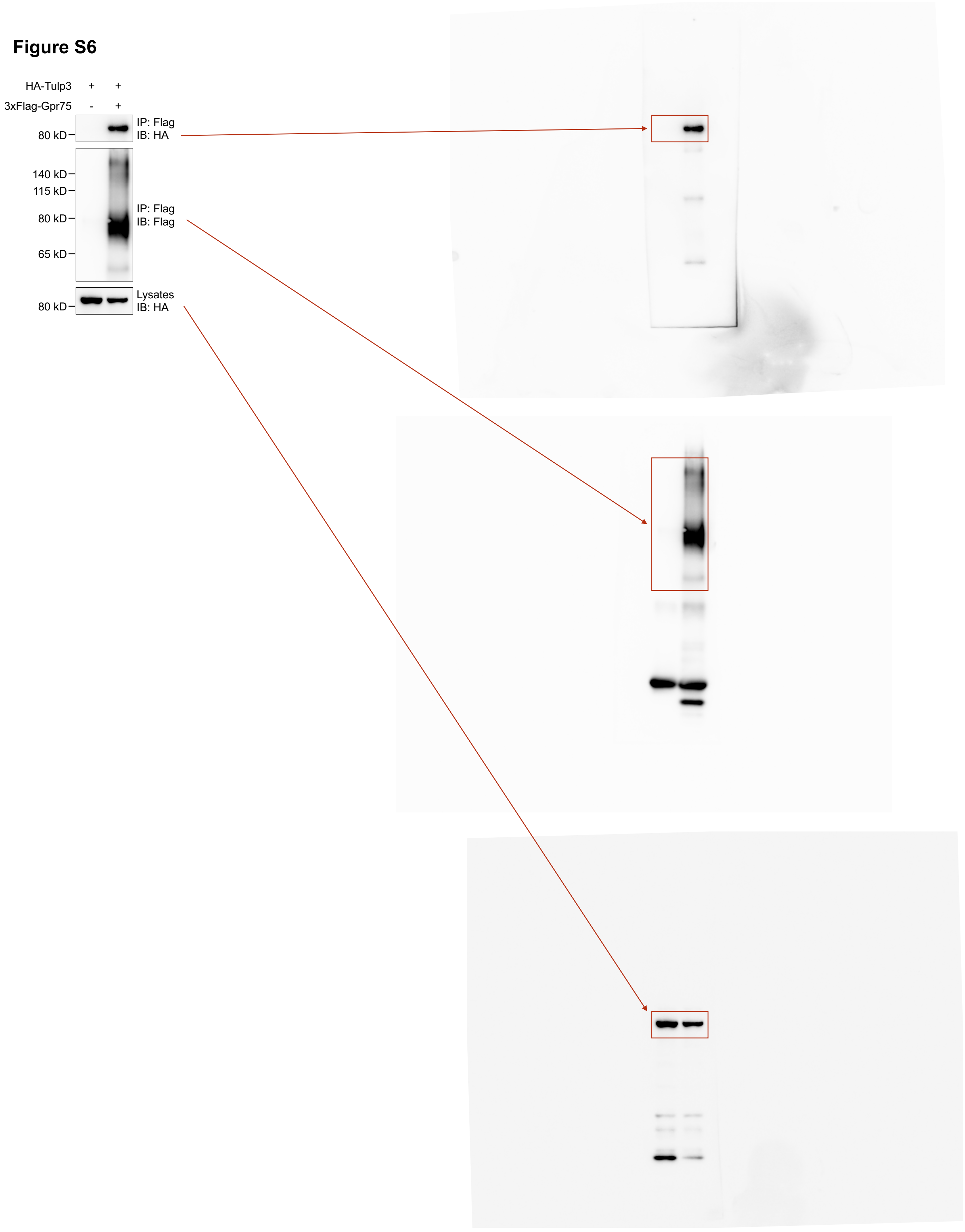

Supplement: Unedited blot and gel images [file jci-134-182121-s140.pdf]
